# Research progress on the role and mechanism of DNA damage repair in germ cell development

**DOI:** 10.3389/fendo.2023.1234280

**Published:** 2023-07-17

**Authors:** Yan Wang, Mengrong Su, Yujie Chen, Xinyu Huang, Lian Ruan, Qizhuang Lv, Li Li

**Affiliations:** ^1^ College of Basic Medical Sciences, China Three Gorges University, Yichang, Hubei, China; ^2^ College of Biology & Pharmacy, Yulin Normal University, Yulin, China

**Keywords:** DNA damage, DNA repair, germ cell, reproduction rate, abnormal DNA damage repair

## Abstract

In the complex and dynamic processes of replication, transcription, and translation of DNA molecules, a large number of replication errors or damage can occur which lead to obstacles in the development process of germ cells and result in a decreased reproductive rate. DNA damage repair has attracted widespread attention due to its important role in the maintenance and regulation of germ cells. This study reports on a systematic review of the role and mechanism of DNA damage repair in germline development. First, the causes, detection methods, and repair methods of DNA damage, and the mechanism of DNA damage repair are summarized. Second, a summary of the causes of abnormal DNA damage repair in germ cells is introduced along with common examples, and the relevant effects of germ cell damage. Third, we introduce the application of drugs related to DNA damage repair in the treatment of reproductive diseases and related surgical treatment of abnormal DNA damage, and summarize various applications of DNA damage repair in germ cells. Finally, a summary and discussion is given of the current deficiencies in DNA damage repair during germ cell development and future research development. The purpose of this paper is to provide researchers engaged in relevant fields with a further systematic understanding of the relevant applications of DNA damage repair in germ cells and to gain inspiration from it to provide new research ideas for related fields.

## Graphical Abstract

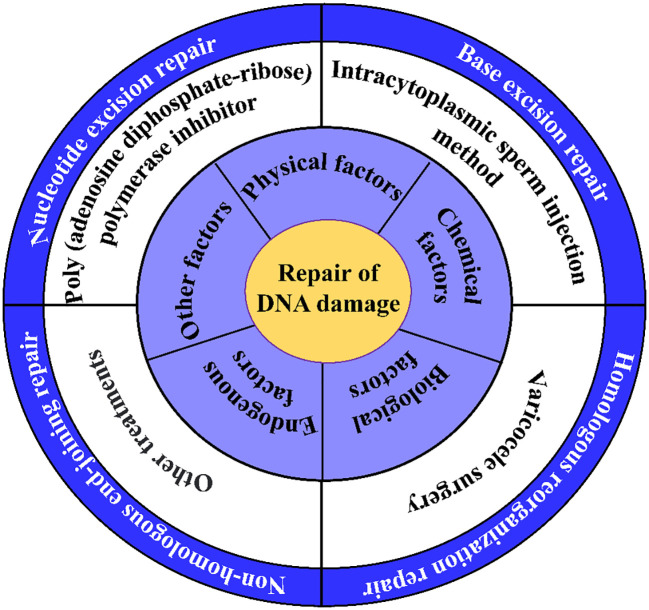

## 1 Introduction

Due to the complexity of their structure and function, DNA molecules are prone to many replication errors and damage during the complex processes of replication, transcription, translation, etc. There are many causes of DNA damage. Ultraviolet irradiation can cause the formation of dimers between thymine or cytosine, X-rays and γ rays may break the single or double strands of DNA, and alkylating agents such as methyl thiomethane may cause strand breaks, etc. Other agents exist which also cause damage by a range of processes. DNA damage leads to obstacles in the development of germ cells, which may lead to reproductive diseases or irreversible genetic diseases of the fetus. This is considered to be closely related to the decline in fertility rate and has attracted much research attention.

DNA damage repair can restore DNA structure through the action of a variety of enzymes so that the molecule can perform its original function. However, repair cannot completely eliminate such damage (1), but only enable the cell to tolerate the damage, allowing the DNA to survive. The damage may manifest under certain conditions during subsequent cell development, but without DNA damage repair, the cell may not survive. For example, the frequency of base mismatches is approximately 10^-1^-10^-2^, but it is reduced to 10^-5^-10^-6^ under the action of DNA replicase. If incorrect nucleotides occur during the replication process, DNA polymerase stops replicating, which then jeopardises the accuracy of germ cells to a large extent. Through DNA damage repair by the cells themselves or artificial intervention, the DNA damage rate is reduced, thus reducing the damage to germ cells caused by DNA. Currently, known repair methods for DNA damage include excision and recombination (2). However, DNA damage repair is rarely used in the clinical treatment of reproductive diseases. The most common treatment methods include drug and surgical therapies, including the use of antioxidants, icariin, and other drugs, and varicocele surgery.

Humans have not yet understood and perfected the function and application of DNA damage repair, despite it having a key role in cancer, genetic disorders, and other diseases. As the fertility rate in some developed countries declines annually, increasing attention has been given to the treatment of reproductive diseases by DNA damage repair, and its related applications in germ cells. Therefore, it is necessary to systematically review recent reports on applications of DNA damage repair in germ cells. First, the causes and repair mechanisms of DNA damage are summarized, including endogenous and exogenous damage, as well as excision repair, recombination repair, and other repair methods. Second, a summary is given of the causes of abnormal DNA damage repair in germ cells, such as increasing age, genital tract infections associated with leukospermia, exposure to xenogeneous organisms, and incomplete and erroneous oocyte repair. Third, we summarize the therapeutic methods for DNA damage repair in reproductive diseases, such as antioxidant and intracytoplasmic sperm injection (ICSI) therapies. Finally, based on the findings and personal understanding, we summarize the shortcomings of DNA damage repair in related applications of germ cells and prospects for the future development of DNA damage repair in reproductive diseases. The purpose of this study was to enable researchers engaged in relevant fields and who have an interest in DNA damage repair and germ cells to have a further systematic understanding of the current literature to gain inspiration from it to promote further development of related fields.

## 2 DNA damage and repair mechanisms

DNA damage is the main cause of cell death and injury due to cells being unable to avoid damage during replication, transcription, and translation. The types of damage are mainly divided into endogenous and exogenous (3). Endogenous damage is caused by spontaneous DNA errors, and its rate of occurrence is extremely high. It includes replication errors, spontaneous chemical changes, and oxidation damage to bases. Exogenous damage is caused by changes in the external environment and includes base loss or modification and chemical bond breakage. These injuries predispose individuals to cancer or cytopathies, particularly of germ cells, which can easily lead to reproductive diseases. However, these damages also involve a series of corresponding repair mechanisms that can minimize the degree of DNA damage.

### 2.1 Cause and mechanism of DNA damage

**Figure 1:**
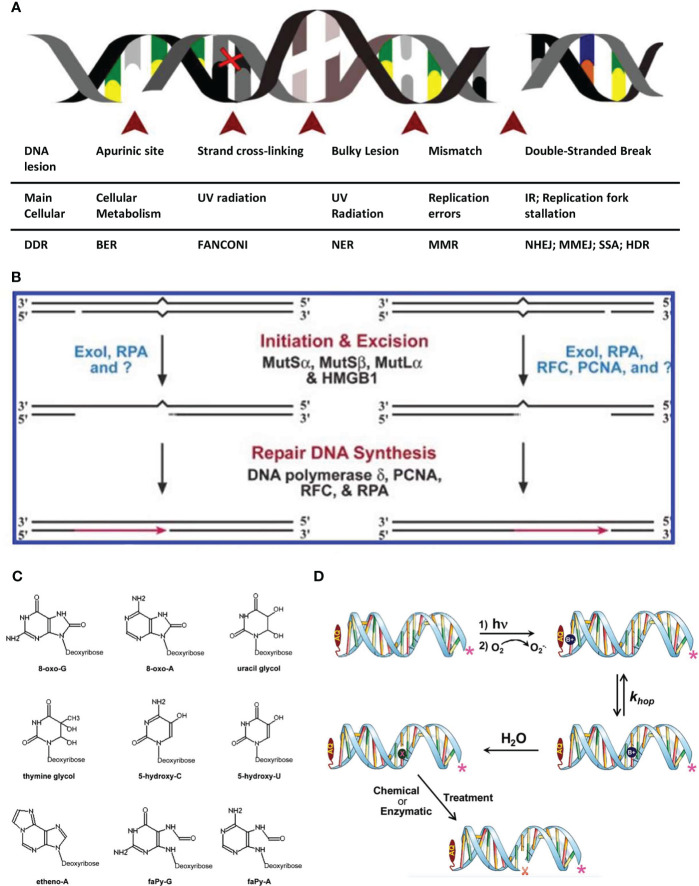
Schematic diagram of common factors causing DNA damage. **(A)** Schematic of the major DNA lesions experienced by cellular genomic DNA: The table below indicates the type of DNA lesion depicted in the figure above, its leading cause and the DNA repair pathway engaged for its resolution (4). **(B)** Human bidirectional mismatch repair *in vitro*. Human mismatch repair *in vitro* can be directed by a strand break located either 3′ or 5′ to the mismatch. Activities that have been implicated in several steps of the reaction are shown. Question marks indicate that unidentified activities may also play significant roles in the reaction (5). **(C)** The most common oxidative DNA lesions. 7,8-dihydro-8-oxo-guanine (8-oxo-G); 7,8-dihydro-8-oxo-adenine (8-oxo-A); uracil glycol; thymine glycol; 5-hydroxycytosine (5-hydroxy-C); 5-hydroxyuracil (5-hydroxy-U); ethenoadenine (etheno-A); 2,6-diamino-4-hydroxy-5-formamidopyrimidine (faPy-G) and 4,6-diamino-5-formamidopyrimidine (faPy-A) (6). **(D)** A schematic representation of the photooxidation of DNA leading to strand cleavage. In the first step, UV light is absorbed by AQ15 forming its excited state, which oxidizes an adjacent nucleobase forming the radical cation (B+•). In a subsequent step, the concomitantly formed anthraquinone radical anion (not shown) reacts with O2 to form superoxide (O2 -•), and in that process the AQ is regenerated. The B+• may hop reversibly through the duplex DNA (with generic rate constant *k*hop) until it is trapped in a chemical reaction with H2O (or another reagent such as O2 -•) 25 resulting in a damaged nucleobase that is symbolized generally as “X”. Subsequent chemical or enzymatic treatment results in strand cleavage at the site of reaction (7).

DNA contains genetic information required for the construction of organisms and is crucial for maintaining an organism’s life. DNA damage refers to the abnormal structural changes in DNA molecules caused by endogenous or exogenous factors and poses a constant threat to cells and organisms (4) (**Figure 1A**). The causes of DNA damage include endogenous and exogenous causes. Harm caused by endogenous damage is more extensive and occurs more frequently than that caused by exogenous damage (8). Errors in DNA replication can cause the production of reactive oxygen species (ROS) and reactive nitrogen species (RNS), both of which are endogenous sources of DNA damage (9). The sources of exogenous factors are more complex and include cosmic radiation, X-rays, and mutagenic chemicals, which cause cells to counteract the daily load of DNA damage, resulting in DNA decay. If damaged DNA is not repaired in sufficient time, it may cause further irreversible damage to cells and organisms.

#### 2.1.1 Endogenous factors

A potential attack on DNA produced by normal cells, namely endogenous damage (8). Endogenous DNA damage is mainly caused by unavoidable oxidation and hydrolysis in normal cells and is an important problem facing aerobic organisms (10) and includes DNA replication errors, spontaneous chemical changes, and oxidative damage of bases. This type of damage manifests in germ cells and can lead to serious reproductive diseases.

##### 2.1.1.1 Replication errors of DNA

DNA replication errors are the most basic stimuli of endogenous DNA damage. Spontaneous errors in replication may result in the presence of an incorrect nucleotide in a newly synthesized DNA molecule, which will produce mismatches or non-complementary base pairs in the DNA structure. If not corrected, these will result in mutations in the next round of DNA replication. In addition, genetic recombination may result in mismatching when heteroduplex intermediates cross the genetic differences between the recombination helices (5) (**Figure 1B**). DNA damage caused by DNA replication errors in germ cells may lead to endometrial cancer. In related studies, it has been found that mutations in the polymerase epsilon catalytic subunit (POLE) gene account for 7%–10% of all gene mutations in endometrial cancer. In the case of this mutation, the proofreading ability of the cell is limited, leading to increased DNA replication errors, which may also lead to messenger RNA and amino acid errors, resulting in structural and functional changes in related proteins. This eventually leads to the development of endometrial cancer (11).

##### 2.1.1.2 Spontaneous chemical changes

The structure of DNA itself is unstable, so the base deoxynucleotides on the DNA strand are prone to depurination, depyrimidine, deamination and other spontaneous chemical changes. In addition, DNA methylation can occur in the body. If the cell does not have an efficient repair system, the accumulation of DNA damage will lead to a greatly increased biological mutation rate. It is a serious threat to human life and health. DNA methylation is an important chromatin modification that widely exists in the promoter region, heterochromatin region and euchromatin region of transcription-inactive genes, and participates in transposon silencing, gene transcription regulation and heterochromatin structure maintenance. For example, Karahan et al. found that methylenetetrahydrofolate reductase (MTHFR) deficiency causes DNA methylation in mouse sperm, and a large amount of DNA methylation loss was observed in both F1 and F2 sperm, and 80% of the sites may be passed on to the offspring. Although MTHFR deletion in the F1 generation has little effect on sperm count and testicular weight, the adverse effects are further aggravated in the F2 generation, resulting in the adverse phenotype of F2 MTHFR deficient males (12). In addition, H19, which contains four introns and five exons, is an important imprinted gene in infertility studies. For example, Vidal et al. detected cervical biopsy specimens from 148 normal women, 48 patients with cervical cancer (CC) and 38 patients with cervical intraepithelial neoplasia (CIN), and found that H19 methylation levels in CIN and CC groups were low and highly expressed, which would increase HPV susceptibility and the risk of invasive CC and CIN (13). Moreover, H19 is easily expressed by methylation in the paternal allele, leading to incomplete fetal development (14).

##### 2.1.1.3 Reactive oxygen species damage bases

ROS are a class of chemically active substances with strong oxidative effects that are widely found in physiological and pathological processes of the body. Oxidative DNA damage is mainly caused by ROS, a complex process involving multiple reactions controlled by a combination of enthalpy, entropy, steric hindrance, and its constituent factors, as well as charge reactions and transport (7) (**Figure 1D**). However, oxidative damage to DNA bases generally occurs on guanine (G), which has the lowest oxidation potential and plays an important role in DNA charge conduction. Under the action of strong oxidative free radicals, the G base is prone to one-electron oxidation, losing one electron to form cationic free radicals (G+·). This generates holes in the DNA chain and conduction along it, making it a potential carrier for charge conduction. At the same time, the reactive activity of cationic free radicals (G+·) is enhanced, which triggers subsequent secondary reactions and leads further to a series of oxidative damages (15), resulting in 8-Oxo G, FAPY-G, and other damage products. Endogenous oxidative damage mainly comes from O_2_^·−^ produced by cellular respiration. This superoxide anion free radical itself is inactive; however, because of the appearance of hydrogen ions, it easily produces H_2_O_2_, which is related to the oxidation of plasma membrane lipids in polyunsaturated acids (16–18) and has a damaging effect on DNA. In addition, oxidative DNA damage is caused by endogenous chemical changes in metabolic reactions and enzyme activities (19). During the normal metabolism of cells, the body is exposed to exogenous chemical carcinogens and irradiation. For example, ROS can be produced under conditions such as toxins (6), ionizing radiation (20–22), and inflammatory reactions (23), leading to various forms of DNA damage, including intra-strand/inter-strand cross-linking, base modification or loss, and strand breaks. In addition, some diseases lead to the production of large amounts of reactive oxygen species (ROS), causing DNA damage. According to relevant reports, a large amount of ROS may be produced in the testicular cells of patients with diabetes, resulting in damage to mitochondrial function and the consumption of a large number of antioxidant enzymes. This results in an imbalance between the generation and clearance of ROS, thus causing lipid peroxidation and DNA damage in biofilms (24). Excessive accumulation of ROS in the body breaks down the body’s antioxidant defense system or exceeds its antioxidant capacity and causes DNA damage. If this damage cannot be repaired correctly in sufficient time, it may cause permanent changes in key genes, leading to cell carcinogenesis, gene mutations, and other conditions. This can cause a certain degree of DNA damage in germ cells (25) (**Figure 1C**). ROS can cause lipid peroxidation in the sperm cell membrane; however, both lipid peroxidation and oxidative stress (OS) can affect sperm motility. The former can induce apoptosis of germ cells and affect the vitelline membrane fusion ability of ovarian cells and sperm, whereas the latter can cause damage to germ cells. Moreover, DNA in the sperm nucleus is one of the most sensitive targets of oxidative stress, mainly producing substances such as 8-hydroxy-deoxyguanosine base adducts (26). Nabil et al. tested the ROS content in the sperm of 19 normal and 39 infertile patients with abnormal sperm morphology and found that the sperm of infertile patients had higher ROS content, particularly in the proportion of sperm with acrosome injury, midsection defects, cytoplasmic droplets, and tail defects. The experimental results clarified that excessive ROS content would affect sperm motility but could not fully elucidate the link between ROS and sperm morphology (27). Koppers et al. conducted further studies and found that lipid peroxidation of unsaturated fatty acids in the sperm membrane causes fatty acids to lose their double bonds, thereby causing the sperm membrane to lose fluidity and integrity (28). However, with the discovery of antioxidants, it is possible to repair sperm DNA damage caused by ROS.

##### 2.1.1.4 Mismatch formed by base tautomerism

Base tautomerism refers to the fact that, without considering the “complementary” chaperone base, the tautomeric mispairing of bases is formed under the interaction of bases. The amino-imino or amino group in the base is ketone-enol or ketone structural interchange and may cause changes in complementary bases when used as templates. The phenomenon of tautomerism can lead to base mismatches, mutations and even genetic damage. Some researchers have used NMR technology to find oscillation mismatches of rG·rU and dG·dT during the relaxation and dispersion of DNA and RNA. Transient homeostasis and low-filling Watson-Crick-like mismatches were stabilized by enol and anionic bases. During the relaxation and dispersion of DNA and RNA, a swing mismatch of rG·rU and dG·dT was found. In addition, these mismatches escaped Watson-Crick’s high-fidelity checkpoint with a probability of 10^-3^ to 10^-5^, indicating that this is a common error in replication and translation. In addition, related research show that tautomeric and anionic bases are widely present in nucleic acids, and that their functional and structural complexities exceed those of typical bases. These tautomers have obvious physical and chemical characteristic requirements, such as highly stable control-transfer conditions (29). In addition, different base tautomerization methods have different mapping preferences, such as thymine and guanine pairing in enolades, imine-cytosine pairing, and adenine preferential pairing (8). In addition, Sambroano et al. used DFT to study three pairs of tautomers and the stability of seven 5-Me Cyt isomers (30). However, there are still no reports on the theoretical study of the kinetic mechanism of enol-amino, keto-amino, and 5-Me Cyt enol-imine tautomerism.

#### 2.1.2 Exogenous factors

Exogenous factors, including physical (31), chemical (32), and biological factors (33), can also cause DNA damage. Common physical factors include ionizing radiation (IR), UV lamp irradiation, and X-rays. Chemical factors include base analogues, base modifiers, and alkylating agents. Biological factors are mainly substances produced by heterogeneous organisms that affect DNA, and most of them are toxins such as aflatoxin. Most exogenous factors cause damage to sperm DNA, but relatively little damage is caused by exogenous factors depending on where and how the ovum itself is fertilized.

##### 2.1.2.1 Physical factors

**Figure 2:**
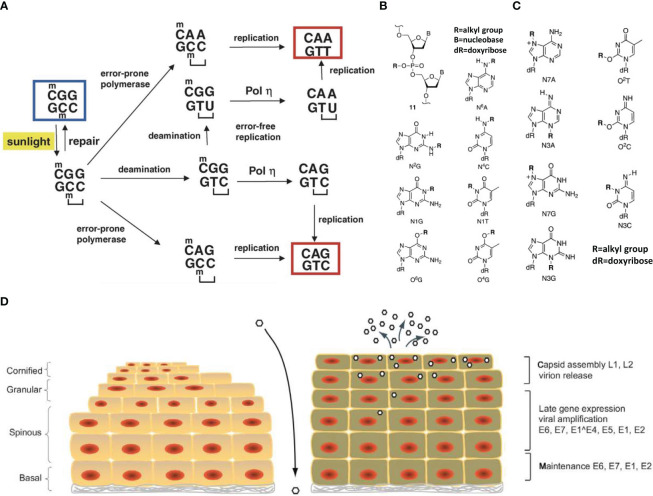
Schematic diagram of DNA damage mechanism. **(A)** Mechanisms of UV mutagenesis at a cyclobutane pyrimidine dimer (CPD). The sequence context shown, 5 -CmCG, is particularly prone to CPD formation by irradiation with sunlight due to the presence of the 5-methylcytosine base (mC). The CPD is indicated by a bracket. Bypass of the CPD by an error-prone DNA polymerase could produce a C to T or CC to TT mutation. Deamination may occur within the CPD and may affect the 5-methylcytosine alone or the 5-methylcytosine and cytosine (double deamination). If the deaminated CPD is bypassed mostly error-free by DNA polymerase, a C to T transition or CC to TT tandem transition mutation will occur. The starting sequence is marked by a blue box. The two mutated sequences, which are eventually produced, are marked by red boxes (35). **(B)** Chemically stable lesions resulting from DNA alkylation (36). **(C)** Chemically labile lesions resulting from DNA alkylation (36). **(D)** Life cycle of human papillomaviruses (34).

Common physical factors that cause DNA damage include infrared (IR) and ultraviolet (UV) radiation. Radiation is mainly divided into IR and non-ionizing radiation, with the most common environmental radiation belonging to the latter, e.g., radio waves, microwave-related electromagnetic radiation, and ultraviolet rays (34). Continuous research has shown that non-infrared radiation sources such as UV radiation (**Figure 2A**) can cause DNA damage in cells (37). IR is mainly composed of X-rays, neutron rays and γ rays.

IR can damage DNA through direct and indirect pathways. In addition, the spectrum of damage induced by IR is similar to that induced by ROS such as thymidine diol, 8-oxoguanidine, and formamide pyrimidine. In addition, IR may cause special single-strand (3) and double-strand breaks. IR not only acts on DNA molecules, but also produces a large amount of ROS. For example, Baulch et al. found that mature sperm cells undergo 45 days of radiation, with some chromosomal changes that are genotoxic for the next three generations (38). Additionally, Tateno et al. found that when sperm were exposed to 4Gy γ and 2Gy radiation, the incidence of chromosome aberration was 35.7% and 25.9%, respectively (39). In addition, Said et al. used 3.2 Gy γ-rays for whole-body irradiation of female rats, and their results showed that granulosa cells in the ovary had increased apoptosis, oxidative damage, and inhibition of cell proliferation, which eventually led to premature ovarian failure (40). Thus, the DNA damage effects of IR on germ cells should not be underestimated. Electromagnetic radiation (EMF) may cause electrons to produce both unquantified and excessive heat which leads to oxidative stress (OS) (41, 42). Although the mechanism of interaction between EMR and biological systems remains unclear, increasing *in vitro* and *in vivo* experimental evidence suggests that EMR may interfere with the oxidative and antioxidant balance of cells, eventually leading to OS. If the OS produced is too strong, it will damage testicular tissue. Iuliis et al. found that exposure to radio-frequency electromagnetic radiation (RF-EMR) may increase ROS content in functional human sperm (43). Human testicular tissue contains Leydig cells and support cells, and the number of Leydig cells is directly related to the production of testosterone. Irradiated sperm may modify the arrangement of microtubules and interrupt the work of mitochondria (44), which can damage the function and morphology of germ cells and even cause serious damage to DNA in germ cells, resulting in defective DNA. Moreover, it may directly affect the DNA damage repair function and cause adverse effects in future generations (45, 46). For example, Houston et al. found that exposure to 1800 MHz radiofrequency (RF) radiation with specific absorption rate (SAR) of 0.15 W/kg for 3 hours caused DNA fragmentation in spermatocyte cell line GC-2 and spermatogonial cell line GC-1. Prolonged exposure to 4 hours may cause oxidative DNA base damage (47). UV radiation is a common form of non-ionizing radiation that causes exogenous DNA damage and is divided into different wavelengths such as UVA (320−400 nm), UVB (280−320 nm), and UVC (200−280 nm). The most common damage induced by UVB and UVC radiation includes cis-cyclobutane pyrimidine dimers (CPD) and pyrimidine-pyrimidine ketone photoproducts (PPs) (35), whereas UVC mainly damages DNA by generating covalent bonds between adjacent pyrimidines. Matsunuma et al. confirmed that DNA damage caused by UV irradiation may induce HBO1 phosphorylation, which promotes CRL4^DDB2^ degradation and regulates cell proliferation (48). At present, there are relatively few studies on the DNA damage caused by UV light in mammalian germ cells, with the research mainly focussing on somatic DNA damage.

##### 2.1.2.2 Chemical factors

The study of DNA damage caused by chemical factors first emerged from research on chemical weapons. Common exogenous chemical factors include base analogues, modifiers, and alkylating agents. Base analogues and modifiers can damage DNA and are often used as mutagens, such as 2-amino-adenine and 5-bromouracil. The basis of damage is due to their structure similarity to that of a base and their entry into the gene sequence instead of the base, thereby interfering with DNA replication. Common alkylation reagents include alkyl sulfates, haloalkanes, nitrosamines, and alkyl sulphonates (**Figures 2B, C**). Alkylation mainly produces chemically stable adducts, such as alkylated adducts at N^2^G, N^4^A, and N^6^A of the outer ring nitrogen atoms, and chemically stable adducts at O^4^-T and O^6^-G produced by oxygen. Among these, the adducts at O^4^-T and O^6^-G have an impact on the Watson-Crick hydrogen-bond surface and can easily cause mutations and miscoding during DNA replication, resulting in DNA damage (36). In addition, the downregulation of gene expression caused by alkylation reagents can affect spermatogenesis and sperm function, and combined cell body injury and chromosomal malformations may occur, causing DNA damage in the sperm. For example, Aguilar-Mahecha et al. studied the effect of cyclophosphamide (an alkylating agent) on male rat germ cells and showed that it can directly or indirectly induce DNA double-strand breaks, which have genotoxic effects on male germ cells (49). In addition, some endocrine disruptors (EDCs), such as nonylphenols, polychlorinated bisphenols, and organochlorine pesticides can affect the regulation of the endocrine system. These EDCs have adverse effects on the development of reproductive organs in humans and other organisms and affect the reproductive and physical health of future generations (50–52). For example, Liu et al. found that exposure to bisphenol A (BPA) in the rare minnow *Gobiocypris rarus* can significantly increase the level of methylation in ovary 7 after exposure, and that 35 dcyp19a1a gene promoter region methylation levels and the gene mRNA expression level had a significant negative correlation (53). The entry and proliferation of primordial germ cells (PGCs) into the genital ridge is characteristic of germ cell production. However, EDCs can affect PGCs proliferation by altering the methylation levels of related genes, further affecting germ cell production.

##### 2.1.2.3 Biological factors

Biological factors that cause DNA damage are mainly toxic substances produced by xenoorganisms, of which aflatoxins are more common. Some RNA viruses and parasites can also cause DNA damage to a certain extent, among which herpes simplex virus types I and II are associated with cervical cancer. After aflatoxins enter the human body through media or directly, they are transformed by microsomal mixed-function oxidase (MFO), which is mainly distributed in the liver. The conversion of aflatoxin B1 in adults and foetuses depends on different substances; in adults, it occurs through the cytochrome P450 enzyme P450 III AY for conversion, and in foetuses it mainly relies on P450 III A6 in the liver for conversion, and the results are converted into AFB1 main metabolite (AFQ1). However, the main active form of AFB1 is generally considered to be the AFB1 epoxide, which may attack oxygen and sulfur heteroatoms and nucleophilic nitrogen in cellular components. They belong to highly reactive substances that may combine with DNA bases and cause DNA damage (54). Hausen found that HSV-II infection can promote the invasion of HPV into cervical cells and cause DNA damage, leading to cervical cancer (55) (**Figure 2D**).

White blood cells have the role of detecting and eliminating viruses. If the large increase in white blood cells in semen is due to bacterial infection of the genitourinary tract, the bacterial infection itself has little effect on DNA. However, white blood cells are prone to produce ROS during the process of fighting against viruses, which may lead to DNA damage. For example, Saleh et al. found that leukocytes may induce sperm DNA damage with cascade amplification effects, while affected patients have significantly increased ROS production rates and reduced sperm motility. They verified, by using single-cell gel electrophoresis (SCGE) and sperm chromatin structure analysis (SCSA), that white blood cells are the main source of ROS production in semen and have a great impact on DNA integrity (56). In addition to bacteria and viruses harming the DNA of germ cells, fungi may also have a certain impact on the DNA of germ cells. For example, Zheng et al. showed that zearalenone can cause irreversible damage to the skeletal structure of germ cells (loss of mitochondria and golgi) and also has a certain impact on DNA replication and transcription, causing DNA damage (57). In addition, some viruses cause inflammation of testicular and epididymal tissues (58, 59), and these excessive inductions trigger ROS production, which can damage the DNA of germ cells and developing sperm.

### 2.2 Methods of DNA damage detection

Even under optimal conditions, DNA undergoes continuous chemical modification. The DNA damage that has been identified thus far primarily encompasses single-strand breaks (SSB), double-strand breaks (DSB), cyclobutane pyrimidine dimers (CPD), 6-4 photoproducts (6-4 PP), and their Dewar valence isomers (60). DNA damage is caused by alkylating agents, aqueous deamination, free radicals, and ROS generated by various photochemical processes including ultraviolet (UV) radiation (61). This significantly affects fundamental biological processes, such as cellular growth, development, metabolism, and heredity, and it may even cause cellular mutations, aging, or death. Therefore, research on DNA damage and its detection methods is of paramount importance. Presently, the commonly used methods for detecting DNA damage include polymerase chain reaction (PCR) (62), comet assay (63) and γ-H2AX detection (64).

#### 2.2.1 Polymerase chain reaction

Polymerase chain reaction (PCR) technology is a nucleic acid amplification technique that simulates the natural processes of DNA replication *in vitro* (65). It involves three basic processes: denaturation, annealing, and extension (66). Over the past few years, this technology has been widely used in biological science. Its outstanding advantage is that DNA can be separated from the organism and its replication can be carried out *ex vivo*. It can also be used to greatly increase the amount of minute DNA, which is of great significance in many research and identification studies. Currently, relevant research has found that the PCR technique can be used to detect DNA damage, due to the amplification phase stopping at the site of where it occurs. Wang et al. developed a PCR-based short interspersed nuclear element (SINE)-mediated detection method that uses the abundance, dispersion, and conservation of SINEs to detect UV-B-induced DNA damage and repair in mammalian genomes (67).

#### 2.2.2 Comet assay

The comet assay, also known as SCGE, is a technique used for the rapid detection of DNA strand breaks at the single-cell level, which allows the measurement of the extent of genetic damage. This technology is primarily used to detect single-stranded breaks, double-stranded breaks, and DNA oxidative damage caused by factors such as UV, ultrasound, and electromagnetic radiation (68). The mechanism of the comet assay involves embedding cells in agarose on a microscope slide, lysing the cells with Triton X-100 and 2.5 M NaCl to remove the cytoplasm and most nuclear proteins, and leaving behind the supercoiled DNA as “nucleoid-like” structures. During electrophoresis, negatively charged DNA fragments migrate toward the anode, but significant movement occurs in the case of DNA damage, which can be observed under a microscope as a “comet tail” extending from the nucleoid. The degree of DNA damage in individual cells can be quantitatively determined by measuring the migration length or optical density of the migrating portion of the DNA. Studies have reported the use of comet assays to assess male sperm DNA integrity and evaluate the quality of sperm genomes. Kumar et al. used alkaline and neutral comet assays to assess DNA damage, and their results showed that sperm DNA damage was higher in men with testicular cancer than in fertile donors (69).

#### 2.2.3 γ-H2AX assay

The γ-H2AX assay is commonly used to detect DNA damage and double-strand breaks (DSBs) and can reflect the extent of DSB damage and repair. This technique has the advantages of high specificity, a short detection cycle, and high sensitivity. The mechanism of detection of DNA damage and repair is as follows. Within a few minutes of DNA damage, γ-H2Ax aggregates at the site of the damage and forms γ -H2Ax focal points. Within half an hour, the number of focal points peaks and guides the repair proteins to repair the damaged DNA. As the content of γ-H2AX is positively correlated with the degree of DNA damage, it can be used as an important biomarker for DNA damage. Lee et al. developed a rapid and high-throughput γ-H2AX assay based on imaging flow cytometry (IFC) to evaluate the repair kinetics of DNA DSBs in irradiated human peripheral blood cells (70). However, γ-H2AX is also generated during DNA replication, leading to apoptosis. Therefore, it is crucial to determine the dynamics, quantity, size, and morphology of γ-H2AX-related foci to distinguish them from actual DNA damage.

#### 2.2.4 High-throughput sequencing

High-throughput sequencing technology, also known as Next generation sequencing (NGS), it is often used to detect a large number of submicroscopic copy number variations (CNVs) in the human genome, including deletion, insertion, amplification, and inversion of single DNA fragments, and the most common is genomic microdeletion or microduplication. In recent years, high-throughput sequencing technology has developed rapidly. Previous research experiments have shown that it has high resolution, can find a large number of human genome copy number variation and other genetic information through a single detection of samples, and has the advantages of rapid diagnosis and high accuracy, which has been widely used in various fields of life science. In organisms, genome integrity is of great significance and is a necessary condition for the normal function of cells and the maintenance of life. Changes in the genome of germ cells can cause related reproductive diseases. Detection of these changes by high-throughput sequencing technology is of great significance for revealing the causes of certain reproductive diseases and for subsequent research on treatment methods. For example, Liu et al. used high-throughput sequencing of sequence tagged sites (STSs) to finally reveal a novel Y chromosome microdeletion associated with nonobstructive azoospermia (71). In addition, Jasmin et al. used NGS to detect the methylation level of the cytidine phosphate-guanine site (CpG)5962 in the L1 genome of high-risk HPV16, demonstrating that the methylation of this site may be a predictive marker for HPV persistent infection, and that there is a correlation between the methylation of specific CpG and high-risk viral load (72).

### 2.3 Methods of DNA damage repair

**Figure 3:**
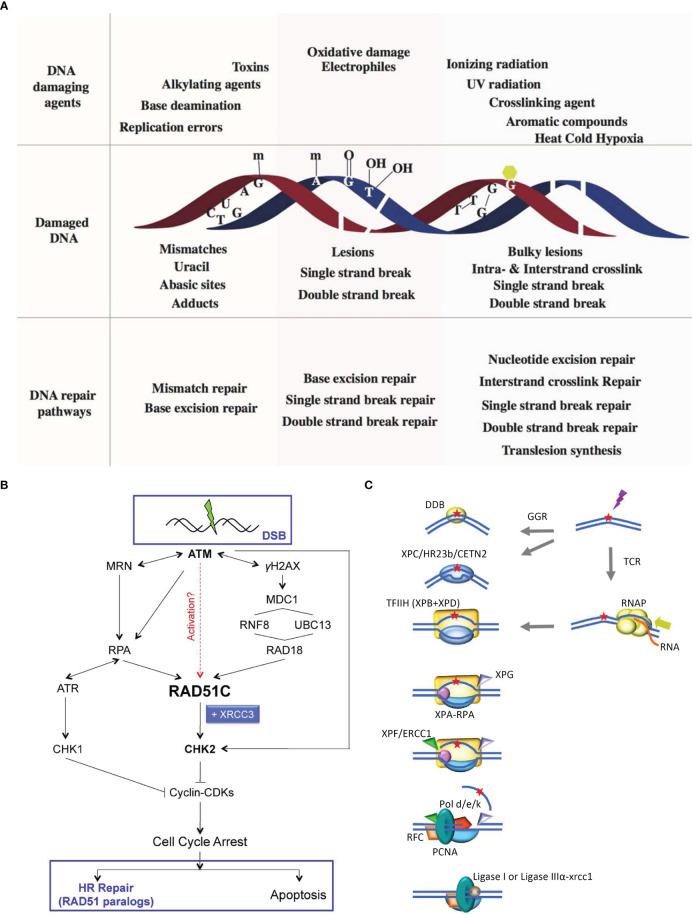
Schematic diagram of DNA damage repair methods. **(A)** Schematic of various DNA damage-induced DNA repair pathways. A variety of DNA damaging agents can induce DNA damage, which becomes substrate for specifific DNA repair pathways. Upper panel shows representative DNA damaging agents: errors from replication, spontaneous base deamination, alkylating agents, toxins, oxidative agents, IR, UV radiation, crosslinking agents, aromatic compounds, and environmental agents such as heat, cold, and hypoxia. Middle panel represents different kinds of damaged DNA: base mismatches (C:T), uracil from deamination of cytosine, an abasic site from the loss of a base from one DNA strand, methylated guanine, methylated adenine, 8-oxo-G lesion, thymine glycols, SSBs, double strand breaks, intrastrand cyclobutane thymine dimers and interstrand guanine crosslinks. The lower panel lists the specifific DNA repair pathways that are instigated to repair DNA damages: MMR corrects replication errors and other base mismatches; BER removes base adducts, uracil, abasic sites and oxidative lesions; SSB repair pathways repairs single stranded breaks in the DNA backbone; DSBR pathway repair double strand breaks; NER removes bulky lesions and intrastrand crosslinks; ICL repair removes interstrand linkages and TLS bypasses intrastrand crosslinks and bulky lesions (3). **(B)** RAD51C links DDR and recombinational DNA repair. Following exposure to IR, RAD51C is recruited to damage-induced nuclear foci in ATM-, NBS1- and RPA-dependent manner. A RAD18-dependent pathway is also required for RAD51C accumulation at the break. Thereafter, RAD51C, possibly in complex with XRCC3, promotes activation of CHK2. ATR is activated in an RPA-dependent manner to phosphorylate CHK1. Active CHK1 and CHK2 inhibit cyclin/CDK complexes leading to cell cycle arrest. Thereafter, the cellular response is dependent on both the extent and the type of DNA damage incurred. If damage is minimal, HR-dependent repair is engaged through largely unknown mechanisms. In response to severe damage, however, cells are eliminated through apoptosis. DNA repair by HR entails activation of RAD51 paralog complexes, both during the early (RAD51 assembly at the sites of damage) and late (HJ branch migration and resolution) steps of the repair reaction (76). **(C)** NER in humans Higher eukaryotes utilize different mechanisms for detecting DNA alterations in actively transcribed genes and in the genome as a whole. In TCR, RNAPII is arrested at a lesion. TCR factors are recruited; the polymerase is removed or backtracked to allow access to TFIIH and other NER repair enzymes. In GGR, a helix distorting lesion or structure can be directly recognized by XPC complexed with hRAD23B and centrin 2 (CETN2). Certain lesions such as CPD, which do not signifificantly destabilize DNA duplexes, are fifirst recognized by DDB2 (XPE) in complex with DDB1, creating a kink that is recognized by XPC. The XPC-hRAD23b-CETN2 complex melts the DNA around the lesion and attracts the multiunit complex TFIIH. TCR and GGR converge; XPB and XPD unwind the DNA to create a∼30-nucleotide bubble. Once the pre-incision complex is assembled, XPA, RPA and XPG are recruited and the XPC complex is released. XPA binds the DNA near the 5’ side of the bubble, and RPA binds the ssDNA opposite the lesion, protecting it from degradation and coordinating excision and repair events. XPG and ERCC1-XPF associate with TFIIH. ERCC1-XPF makes the fifirst incision, and repair synthesis proceeds for several nucleotides displacing the damaged strand; XPG incises the 3 single/double strand junction, and ligase I or ligase III-XRCC1 seal the DNA (73).

During complex processes, such as DNA replication, transcription, and translation, and due to the continuous threat of metabolic byproducts and environmental factors, DNA is highly susceptible to damage. Fortunately, in response to this, biological organisms have evolved mechanisms for preventing and repairing DNA damage (73). The primary DNA repair methods include excision (74) and recombination repair (HR) (75). Excision repair can be classified as either base excision repair (BER) or nucleotide excision repair (NER), while HR can be classified as either homologous recombination repair (HRR) or nonhomologous recombination repair (HRD). These repair mechanisms are active at different stages of cell growth, allowing cells to promptly repair DNA damage and play a significant role in repairing damage in germ cells (3) (**Figure 3A**).

#### 2.3.1 Excision repair

Excision repair (ER) is a DNA damage repair pathway present universally in living organisms. It is commonly used to correct a diverse range of DNA damages, such as oxidative damage, alkylation, and deamination. ER is primarily classified into two types, NER and BER, both of which are major pathways for DNA damage repair. Genetic polymorphisms have been studied extensively. The primary difference between NER and BER lies in the mechanisms for identifying the damage site. BER directly recognizes the damaged base, while NER identifies the distortion of the DNA double helix structure caused by the damage but does not recognize the specific lesions.

NER is a crucial mechanism in cellular DNA damage repair. Once a damaged DNA molecule is identified, NER creates dual incisions on either side of the damaged section, thereby removing damaged DNA fragments. The resulting gap is filled with replicative machinery (**Figure 3C**). NER is mainly used to repair large amounts of DNA damage such as pyrimidine dimers produced by ultraviolet irradiation, other radiation-induced damage, and large chemical adducts. The advantage of NER is that it does not require the specificity of DNA damage sites to activate NER enzymes, and NER has wide coverage. Mechanisms related to nucleotide excision repair are present in both the somatic and germ cells. For example, Roger et al. found that when the nucleotide excision repair pathway is compromised, global hypomethylation and DNA damage can promote genomic instability, ultimately leading to mutations in male germ cells that are subsequently passed on to offspring (77).

Base excision repair (BER) can remove the incorrectly twisted helix DNA damage structure. It is mainly used to repair small DNA base damages that do not seriously affect the double-helix structure of the DNA. In most cases, BER repairs endogenous DNA damage by deamination, oxidative damage, and alkylation. According to the relevant data, BER is a highly conserved repair system covering a wide range (78), and there are five main enzymatic reactions in the repair process. In the repair mechanism of BER, DNA glycosidase recognizes faulty or damaged substrate bases and produces base site intermediates, which are then excised by purine-free/acymidine-free (AP) endonucleases. They are used by lyases or phosphodiesterases to remove the remaining sugar fragments. Finally, DNA polymerase fills the gaps left by the removal and repair of damaged DNA bases (79, 80). Excision repair is widely used in germ cells. For example, 8-hydroxy 2’oxoguanine (8OHdG) is a base admixture produced by sperm when subjected to excessive ROS to produce OS. 8-oxyguanine glycosylase 1 (OGG1) cleaves the 8OhdG residues and produces base sites. The apyrimidine endonuclease then cleaves the main strand of the DNA and inserts unmodified nucleotides into the egg. DNA base excision repair in sperm is different from that in egg cells because there is no pyrimidine-free endonuclease 1 in sperm and the repair of pyrimidine-free sites produced by OGG1 occurs mainly during the S phase of meiosis (81).

#### 2.3.2 Recombination repair

Recombination repair is a common double-stranded DNA damage repair pathway that mainly repairs damaged and mismatched bases of various structures and includes homologous recombination repair (HR) and nonhomologous recombination repair (NHEJ). These two pathways treat the ends of broken DNA in different ways and repair the pathways (76). HR can reconnect the end of DNA only when the DNA sequence is homologous (82) and relies on homologous chromosomes to guide the correction of damaged DNA. In contrast, NHEJ directly connects the broken double-stranded DNA to achieve the purpose of repair, resulting in repair of HR that is more accurate. HR and NHEJ pathways are error-free DNA damage repair mechanisms widely existing in most types of cells and play a very important role in maintaining the stability of genomic information in organisms.

HR requires sequence homology, and its central activity is controlled by the RAD51 protein (83), which catalyzes the capture of DNA double-strand break products and helps the break end penetrate sister chromatids or homologous chromosomes, known as DNA homologous sequences, to ensure the fidelity of DNA repair. Excision is mediated by CtIP, EXO1, DNA2, BLM, and the MRE11-Rad50-NbS1 complex, whereas single-stranded DNA overhangs are enveloped by heterologous replication protein A. ATM phosphorylation activates CHK1 kinase and other effector proteins (**Figure 3B**). The key proteins in HR, BRCA1 and BRCA2, are important cancer suppressors. If these two proteins are absent, the rate of intracellular homologous recombination is greatly reduced and the cell’s sensitivity to IR is increased, leading to an increased risk of ovarian and breast cancers. For example, Bertelsen et al. identified 636 patients with advanced ovarian, cervical, and other cancers, who had high-frequency pathogenic mutations in the homologous recombinant repair gene in DNA repair, by using germline mutations with whole-exome sequencing (84).

NHEJ refers to the direct repair of broken ends through the action of protein-DNA complexes without the need for an homologous template. The principle of NHEJ is to rejoin damaged genes by degradation; thus, it can be used to insert or delete non-specific fragments to achieve the intended gene segment repair. This repair process relies on the DNA-dependent protein kinase (DNA-PK) holoenzyme, as well as the DNALIG4-XLFXRCC4 complex. For example, Cinzia et al. found that cisplatin-based chemotherapy for germ cell tumors (TGCT) has unique sensitivity, and the mechanism of resistance produced by its treatment is largely unknown. They studied the mechanism by which cisplatin acquired resistance, using the TGCT cell line as a model. The results demonstrate that resistance develops through the selection of repair pathways that regulate DNA, i.e., in resistant cells, NHEJ double-strand breaks are inhibited by decreased expression of TP53 binding protein 1 (53BP1) and DNA-dependent protein kinases (DNA-PKcs), while cisplatin-induced DNA damage can be effectively repaired by homologous recombination (85).

## 3 Causes and effects of abnormal DNA damage repair in germ cells

Germ cells mainly include sperm, eggs, spermatogonia, and oogonia. Owing to the different processes of sperm and egg formation before fertilization, the possibility of DNA damage in the egg cells of the female body is significantly less than that in male sperm. The ovum is vulnerable to endogenous damage, whereas the sperm is vulnerable to both endogenous and exogenous damage. Various endogenous insults such as DNA replication errors, spontaneous chemical changes, ROS-damaged bases and tautomer mismatching can lead to DNA damage in eggs, but the eggs can repair the damaged DNA through the HR pathway. At present, comet assay can be used to detect DNA damage in egg cells, and drugs such as poly (ADP-ribose) polymerase (PARP) inhibitors can be used to repair DNA single-strand damage in egg cells. In addition to endogenous damage, exogenous factors such as ionizing radiation, UV irradiation, base analogues and alkylating agents are prone to cause sperm DNA damage. The DNA damage repair mechanisms in males mainly include nucleotide excision repair, basal excision repair, and recombinant repair (81). During sperm development, sperm DNA damage can be identified by comet assay, γ-H2AX assay and high-throughput sequencing, and can be repaired by icariin, antioxidants and other treatments. DNA damage and abnormal DNA damage repair may lead to structural and functional defects or changes in male and female germ cells and may even lead to cancer or other reproductive diseases. Therefore, it is important to understand the causes and effects of abnormal DNA damage repair in germ cells.

### 3.1 Causes of abnormal repair of DNA damage

Sperm DNA damage and repair abnormalities were influenced more by exogenous factors, whereas egg DNA damage and repair abnormalities were influenced more by endogenous factors. The widely recognized sources of sperm DNA damage are mainly ROS, sperm chromatin packaging, and cell apoptosis, in which ROS and cell apoptosis are related to sperm DNA damage. The exogenous sources of ROS are mainly related to increasingage, smoking, herbicides, and double-stranded DNA breaks. High concentrations of ROS and their resulting OS can lead to the loss of sperm motility and DNA damage. Additionally, factors such as age, reproductive tract infections related to leukocytospermia (86), exposure to xenogeneic organisms (87), environmental pollution, and smoking (88, 89) can cause DNA damage or repair abnormalities in germ cells (90).

#### 3.1.1 Growth of age

It is widely accepted that male and female reproductive systems undergo approximately equal numbers of mitoses before reaching sexual maturity. Additionally, during mitosis, the rate of DNA damage in the sperm is higher than that in the ova, and the main cause of DNA damage resulting from reproductive cell aging is DNA replication errors (90).

According to relevant research, there is a positive correlation between the age of the father and the incidence of autosomal dominant genetic diseases such as Marfan syndrome and achondroplasia. These diseases are generally caused by single nucleotide variations and increasing the age of the father can also lead to cumulative accumulation of DNA damage in reproductive cells, resulting in declining fertility. A man’s sperm is produced in a different way than a woman’s ovum. After male sexual maturation, stem cells undergo continuous mitotic division. During this process, DNA is prone to random mutations, mismatches, and repair failures. Additionally, there was a significant correlation between age and the degree of DNA damage and a direct link between age and DNA breakage (91). For example, Wyrobek et al. found that the sperm DNA fragmentation index (DFI) increased gradually with age between 20 and 80 years, with a progressive upward trend (92). Templado et al. used multicolor fluorescence hybridization (MFISH) to find that all sperm in older men had autosomal duplication and deletion, which constituted structure abnormalities. Both rates were significantly higher than those in young men (6.6% and 4.9%, respectively). The experimental results also confirmed that the recurrence rate exceeded the deletion rate regardless of age, with a ratio of 2:1 (93). Additionally, with increasing age, there is a tendency for the accumulation of ROS, which are necessary for normal sperm metabolism and function. However, excessive ROS can bind to DNA to induce double-strand breaks and damage sperm DNA and proteins (94–96).

The effect of age on the ovum is relatively small. Currently, the main reasons for age-related DNA damage in ova are DNA replication errors and the accumulation of free radicals. As age increases, cell detection and repair capabilities decrease. Ova development differs from that of sperm, and egg development continues from the embryonic period until adolescence. Subsequently, ova consistently exist inside a woman’s body. However, because genetic mutations are random and universal, mutations, replication errors, and inadequate repair of some ovaries are inevitable, and the likelihood of such situations occurring is relatively low. Additionally, the degree of methylation in the ova increases with increasing age (97).

#### 3.1.2 Reproductive tract infection associated with leukocytospermia

Leukocytes have the ability to monitor and engulf abnormal sperm and are widely present in the semen (98, 99). Leukospermia is diagnosed when the leukocyte count in the semen exceeds 1 × 10^6^/ml. Leukospermia indicates the presence of infection or inflammation in the urine and reproductive organs of males. Males with leukospermia exhibited significantly higher levels of DNA damage in their semen than normal males without this condition (100).

Semen white blood cells are produced in the epididymis and secondary gonads (101). Leukocytospermia is not only related to the content of white blood cells but also to the activation status of white blood cells in semen (102). Because of the tightness of the blood-testis barrier, the proliferation of white blood cells does not affect the production of sperm in the testis. The decrease in sperm quantity is related to the secretion of accessory sex glands stimulated by the proliferation of white blood cells, ultimately leading to diluted semen and fewer sperm. In addition, a large number of white blood cells infiltrate the prostate epithelium and epididymis, resulting in gonadal dysfunction which affects sperm maturation in the reproductive tract. Additionally, it has been found in continued research that white blood cells in semen can produce a large amount of ROS during phagocytosis. Sperm in semen are protected by antioxidants (103), but ROS content in the sperm exceeding the protective level of semen may cause OS to occur, leading to DNA damage (104). For example, Collodel et al. discovered that when the reproductive tract is infected, there is an increase in malondialdehyde levels in the semen, accompanied by a decrease in sperm vitality and an increase in the number of apoptotic spermatogenic cells. Malondialdehyde is considered a marker of OS in sperm cell membrane lipids, suggesting that sperm apoptosis may be related to the increased ROS levels caused by infection (105).

#### 3.1.3 Exposure to xenogeneic organisms

Semen can serve as a carrier of viruses, rendering the male reproductive system vulnerable to viral infections. At least 27 types of viruses have been reported to use semen as a host, such as herpes simplex virus, human T-cell lymphotropic virus, human immunodeficiency virus (HIV), mumps virus, severe acute respiratory syndrome coronavirus 2 (SARS-CoV-2), Ebola virus, and adenovirus (106, 107). However, some viruses such as coxsackievirus, Zika virus, and influenza virus do not directly alter DNA in reproductive cells (108). These viruses typically target the testes and epididymis, and cause DNA damage in reproductive cells by accumulating cytotoxins or inducing OS (109–111). In addition, many researchers believe that DNA damage caused by bacterial infections, such as those from Chlamydia and Mycoplasma, is due to an increase in white blood cells in the reproductive tract, which leads to an increase in ROS and ultimately causes DNA damage in sperm. For example, Gallegos et al. observed that Chlamydia infection significantly decreased sperm motility and increased DFI, which directly and adversely affected male fertility (112).

#### 3.1.4 Environmental pollution and smoking

With developments in science, technology, and industry, environmental pollution has become a global concern, posing a threat to animal and human health. Common pesticide pollutants include industrial production waste, antineoplastic drugs (113), and chemicals (114). Therefore, the impact of these pollutants on living organisms and germ cells cannot be underestimated. In recent years, increasing evidence has shown that smoking can cause DNA damage to germ cells and has confirmed that cigarette smoke itself has an inductive effect on DNA damage, which is related to a decrease in sperm count and sperm motility and an increase in DNA damage rate in germ cells.

Environmental pollution affects germ cells mainly by affecting sperm development, inducing germ cells to produce ROS, and inducing the degeneration of the spermatogenic epithelium. The ROS produced can damage sperm DNA (115–117). The four mechanisms of air pollution-induced adverse effects on the male reproductive system are endocrine disruption, DNA mutations caused by blood-testis barrier disruption, epigenetic modifications, and ROS induction. Interference with endocrine mechanisms may cause hormone secretion disorders, thereby interfering with sperm activity and causing DNA damage to the sperm. After the blood-testis barrier is destroyed, pollutants in the air or environment may cause an immune response near the testis, resulting in an inflammatory response that damages the DNA of germ cells. Epigenetic modifications can result in DNA methylation and RNA or protein modification, which have an impact on gene expression during various developmental processes in sperm. The production of ROS does not cause significant damage to DNA in a specific amount; however, excessive ROS may trigger OS, causing DNA damage. Therefore, these four mechanisms may cause DNA damage in sperm. For example, Zhou et al. used multivariate regression analyses and mixed-effects models to study the associations between exposure to PM_10_, PM_10-2.5_, and PM_2.5_, semen quality, sperm DNA fragmentation, and serum reproductive hormones. The results of this study showed that exposure to PM_10_ and PM_10-2.5_, but not PM_2.5_, is a risk factor for producing semen of poor quality (118).

In addition to environmental pollution, cigarettes pose a threat to germ cells. Some studies have shown that the rate of DNA damage in the sperm of smokers is significantly higher than that in nonsmokers using SCSA and terminal deoxynucleotidyl transferase dUTP nick-end labeling (TUNEL). Smoking can directly induce DNA strand breaks, chromosomal aberrations, oxidative DNA damage, and changes in gene expression. Smoking increases the baseline frequency of sperm DNA strand breaks by approximately 10%, whereas benzo[a]pyrene from cigarette smoke crosses the blood-testis barrier and induces sperm DNA damage that is ultimately transmitted to the genome of the unborn offspring. When studying the mechanism of benzo[a]pyrene damage in mice, some scholars have found that the DNA damage induced in progeny is not repaired, whereas DNA damage in spermatogonial stem cells can be repaired, and the benzo[a]pyrene-7, 8-diol-9, 10-epoxide adduct can be transmitted to the offspring together with sperm. In addition, the content of benzo[a]pyrene DNA adducts was also significantly increased in the sperm of smokers, suggesting that unrepaired sperm DNA damage may lead to genetic instability in the offspring (119). At present, relevant studies have found that the concentration of white blood cells in the semen of smokers is significantly higher than that of non-smokers, but the mechanism of this occurrence is still unclear. Further research is needed, but most scholars generally believe that it may be due to the metabolites of cigarette smoke causing damage to the male reproductive system. This may cause an increase in white blood cells and DNA damage. An increase in leukocyte concentration may induce ROS production, increase OS in mature or developing sperm, and eventually lead to DNA damage in germ cells. At the same time, the antioxidant levels in the seminal plasma of smokers were lower than those in non-smokers (100).

#### 3.1.5 Poor development of sperm

The occurrence of most male infertility is due to low sperm count and/or poor sperm quality (120–122). With the continuous progress in detection technology, many reasons for male infertility in the past are now traceable. Infertility may be caused by a variety of factors such as poor sperm development, ROS, and malnutrition. Male infertility is a global issue that affects families and emotional relationships between couples. Consequently, male infertility has garnered increasing attention from researchers in related fields (123). Sperm DNA integrity is closely associated with male fertility. Compared to healthy men, male patients with infertility have higher sperm DFI and a higher degree of sperm DNA damage. For example, Varshini et al. showed that the average incidence of sperm DNA damage in patients with normal semen parameters was less than 10%. In contrast, patients with oligoasthenoteratozoospermia (OAT), asthenozoospermia (AZS), oligozoospermia (OZ), severe oligozoospermia (SOZ), and necrozoospermia (NZ) showed significantly increased levels of sperm DNA damage (P < 0.001) (124). Furthermore, Simon et al. found that compared to *in vitro* fertilization (IVF), male patients with high DFI can achieve higher pregnancy rates through intracytoplasmic sperm injection (ICSI), but with no significant improvement in embryo quality and miscarriage rate. Although ICSI can improve the adverse effects of sperm DNA damage to some extent, there is still the possibility of injecting sperm with DNA damage into the ova (125).

#### 3.1.6 Incomplete and incorrect oocyte repair

Oocyte integrity and subsequent development are strongly associated with DNA damage repair mechanisms. The rate of DNA damage at checkpoints in pre-meiosis I oocytes increases significantly; however, the mechanism remains unclear and requires further study. It is widely recognized that during DNA damage repair in germ cells, oocytes first induce mild DNA damage during the lengthy meiotic M phase and then repair the damage in the later stages of the cell cycle. This can make the DNA repair process more selectively advantageous (126). The DNA double-strand repair mechanism and other DNA damage repair mechanisms play important roles in maintaining the DNA of follicular cells (127, 128). In addition, the range and supply of follicles are limited, the detection and repair of DNA are both essential for the survival of germ cells, and incomplete and erroneous oocyte repair will lead to abnormal DNA damage repair. Aging may also reduce the ability of DNA detection and repair of germ cell DNA (129). Abnormalities in the DNA double-strand break repair system may hinder normal oocyte development and cause oocyte death (130). In the process of prenatal ovarian development, when the DNA of germ cells is damaged, the cells stimulate the classical DNA apoptosis mechanism to induce apoptosis. The mechanism of self-apoptosis in postpartum ovaries is not clear, but it is a way to remove DNA-damaged germ cells to deal with DNA damage or DNA damage repair abnormalities.

At present, obesity has become one of the common concerns of the world. Some studies have found that maternal obesity can cause chronic ovarian inflammation and decreased oocyte quality, leading to adverse reproductive outcomes, and can lead to the accumulation of maternal DNA damage. However, the mechanisms by which maternal obesity impairs embryonic development and offspring health remain unclear, and the strategies to improve the adverse reproductive outcomes of maternal obesity have not been well studied (131). For example, Wang et al. investigated the effects of nicotinamide mononucleotide (NMN) on reproductive performance and oocyte quality in obese mice and the underlying mechanisms. The expressions of ovarian development - and inflammation-related genes Lhx8, Bmp4, Adgre1, Ccl2, Tnf-α, Gal-3, Clec10a and IL-10 as well as Bax and Sod1 in oocytes were detected by real-time quantitative PCR (RT-qpcr). The results found that NMN partially improved oocyte quality by restoring actin dynamics and mitochondrial function, reducing DNA damage, meiotic defects and ROS levels, and lipid droplet distribution in oocytes of high fat diet (HFD) mice (132).

#### 3.1.7 Herbicides

Herbicides play a crucial role in modern agriculture, and they have a positive effect on the control of crop weeds. However, due to their impact on the reproductive and developmental functions of humans and mammals, they have attracted more and more attention from relevant researchers. At present, herbicides are widely used in different countries, which cause serious pollution to the environment and threaten human health seriously through the food chain. Herbicides can not only induce cell cycle disorders, but also have a high risk of inhalation in people who are close to herbicides. The exposure concentration of herbicides during spraying and handling can cause more serious damage to the cell cycle. This is associated with changes in the expression of genes involved in subsequent spermatogenesis, induction of ROS production or alterations in the blood-testis barrier, among others. Glyphosate is one of the most commonly used broad-spectrum herbicides in the world, which can reduce sperm motility and cause sperm DNA damage. “For example, Avdatek et al. studied the effects of glyphoate (GLF) on the reproduction of 28 male Wistar rats and evaluated the protective effects of resveratrol (RES). The results showed that GLF administration reduced sperm motility, sperm plasma membrane integrity, glutathione level, and lecithin content, and that in the GLF-treated group, DNA damage, abnormal sperm rates, and high levels of propylene glycol were detected. The results also demonstrated that RES protected the rat testis against sperm and DNA damage (133).

#### 3.1.8 Insecticide

Insecticides are an important part of pesticides. The common pesticides are mainly divided into four categories: organochlorines, organophosphorus, pyrethroids and carbamates. Insecticides can cause DNA damage in male germ cells. For example, most organophosphorus insecticides can embed the helical double strand of DNA, form chimeras with the bases in the DNA molecule, and cause changes in the chemical structure of the bases, thereby causing DNA damage. Pesticide pesticides can also cause male reproductive toxicity by affecting the structure and number of chromosomes in male germ cells. For example, De Jager et al. found that organochlorine pesticides can lead to chromatin condensation in male sperm and an increase in the proportion of sperm with Y chromosomes in semen (134). Meanwhile, pesticides disturbed the methylation levels of reproductive related genes in F0 generation males. For example, Xia et al. found that fenvalerate could affect the reproductive hormone level and reproductive related indicators of F1 generation by interfering with the methylation degree of CP17A1, NR5A1, PGRC1, Ace, Foxo3a, Ptgfrn and other functional genes of genomic DNA in sperm of F0 generation. It has negative effects on the reproductive development of the offspring (135). In addition, pesticides can induce the occurrence of OS. p,p’-DDE can reduce the activities of superoxide dismutase and glutathione peroxidase, thereby inducing oxidative stress, inhibiting the expression of phosphatidylphosphatidylglutathione peroxidase in the male testis, activating the mitochondrial apoptosis pathway of spermatogenic cells in the male testis, and hindering normal spermatogenesis. For example, Albasher et al. found that the organophosphorus pesticide chlorpyrifos down-regulates the mRNA expression of superoxide dismutase and glutathione peroxidase by down-regulating the Nrf2/ARE pathway (136).

### 3.2 Effects of abnormal DNA damage repair on germ cells

DNA damage in germ cells and abnormal DNA repair mechanisms can lead to infertility or sterility. This can result in reproductive disorders, such as oligozoospermia (OZ) or azoospermia (137), sperm morphological abnormalities (138), and asthenozoospermia (139), and potentially impact the fertility of future generations. DNA damage and repair abnormalities in oocytes can lead to reproductive disorders such as polycystic ovary syndrome (PCOS) (140) and primary ovarian insufficiency (POI) (141) (**Table 1**).

**Table 1:** Mechanisms and treatments of DNA damage repair during germ cell development.

Effects of DNA damage repair abnormalities on germ cells	Mechanisms	Treatments	References
Sperm malformation (A. Macrozoospermia, B. Globozoospermia)	A. Macrozoospermia a. Aurora kinase C gene mutation causes incomplete meiosis, resulting in polyploidy in gametes and a large number of DNA double-strand breaks. Delayed or abnormal repair of DNA damage leads to abnormal synthesis of related proteins, eventually leading to macrospermia. B. Globozoospermia a. Mutations or deletions in SPATA16, PICK1, and DPY19L2 can cause DNA damage in sperm, and abnormal DNA damage repair can interfere with protein synthesis and meiosis during spermatogenesis, leading to spherozoospermia and other abnormalities.	Icariin, Intracytoplasmic sperm injection, Varicocele surgery, Antioxidant	(142, 143)
Oligozoospermia or azoospermia	a. Microdeletions in the three non-overlapping regions of AZF a-b-c on the Y chromosome of male sperm resulting in oligospermia or azoospermia due to delayed or abnormal repair of the lesions.	Icariin, Intracytoplasmic sperm injection, Varicocele surgery, Antioxidant	(144–146)
Asthenozoospermia	a. Sperm mitochondria undergo severe morphological changes and subcellular reorganization, and the replication and subcellular organization of mitochondrial DNA cannot be precisely controlled during spermatogenesis; the inability to maintain an appropriate number of mitochondria also affects sperm motility.	Icariin, Intracytoplasmic sperm injection, Varicocele surgery, Antioxidant	(147, 148)
Polycystic ovary syndrome	a. Exposure to environmental endocrine disruptors (EED) during embryonic development can cause hypothalamic-pituitary-ovarian axis dysfunction or changes in the expression of key developmental genes during embryo development, disrupt the body’s hormone levels and endocrine metabolic activities, and induce PCOS-like phenotypes in the offspring. and transcription of pro-inflammatory cytokines leads to an inflammatory response that aggravates PCOS.	Antioxidant	(149, 150)
Primary ovarian insufficiency	a. X chromosome short arm (Xp) deletion in POI patients often involves Xp11.2~p22.1 proximal to the short arm, and most of the equilibrium translocation break points are distributed in the POF2 (Xq13~q21) region, and the deletion and rupture of the POF1 (Xq26~q28) and POF2 (Xq13~q21) regions will lead to ovarian dysfunction.	Intracytoplasmic sperm injection, Antioxidant	(151–153)

#### 3.2.1 Sperm

Spermatogenesis refers to the process by which sperm are produced from spermatogonial cells and commences during sexual maturation. The sperm formation process includes three phases: spermatogonia, meiosis, and spermiogenesis. The duration of the three phases is approximately the same (154). According to related studies, there are certain difficulties in achieving natural conception when the DNA damage rate of sperm is between 30% and 50%. There are two main mechanisms for sperm to repair DNA damage, one is direct repair through DNA repair enzymes, and the other is indirect repair through DNA damage detection and signal transduction pathways to activate DNA repair pathways. Specifically, direct repair methods include base excision repair and mismatch repair. For example, direct repair is accomplished by breaking damaged bases in DNA strands by base excision repair enzymes, followed by filling normal bases and joining strands. Indirect repair methods include non-homologous end joining (NHEJ) and homologous recombination repair (HR). In the event of DNA breaks, sperm can perform indirect repair by detecting the damage signal and activating the relevant genes and signaling pathways. Abnormal repair of DNA damage in sperm severely affects their reproductive potential, potentially increasing the risk of foetal miscarriage or reducing the success and implantation rates of artificial insemination. This may have an impact on the fertility of future generations.

##### 3.2.1.1 Sperm malformation

In patients with abnormal spermatogenesis, failure to repair DNA breaks results in altered sperm maturity, chromatin packaging defects, and condensation in the ejaculation, which manifests as oligospermia or teratospermia, which is characterized by abnormal sperm morphology in more than 85% of the sperm. When sperm is deformed and DNA is damaged, the cell itself mainly repairs two different types of repair mechanisms: single-strand breaks and double-strand breaks. The former refers to the presence of only one strand break in the DNA strand. The damage is caused by finding an identical DNA sequence at the other end of the DNA strand, and through a series of enzymes, The same sequence is inserted into the broken DNA strand to complete the repair. While the latter requires more complex mechanisms to accomplish repair, mainly including non-homologous end joining (NHEJ) and homologous recombination (HR). In these two repair mechanisms, a variety of enzymes are involved, including DNA polymerase, DNA exonuclease, DNA ligase, etc., which cooperate to complete the repair of DNA. It should be noted that sperm need to mature in a very special environment, DNA damage will have adverse effects on its survival and development, so timely and effective DNA damage repair ability is crucial for its development. Teratospermia is considered monotypic when all sperm exhibit unique abnormalities. Currently, two types of monotypic teratospermia are known, namely macrozoospermia and spherozoospermia.

Macrozoospermia, also known as macrospermia head syndrome, is characterized by enlarged and irregular sperm heads in semen accompanied by an abnormally high number of flagella. Studies on sperm DNA fragmentation in some patients have shown that the DNA fragmentation index of patients with macrospermia is increased compared with normal men (142). Aurora kinase C (AURKC) gene is a key factor in meiosis, especially in spermatogenesis, and is closely related to the occurrence of macrospermia. Therefore, AURKC gene is of great significance in revealing the molecular pathogenesis of macrospermia. Globozoospermia, also known as round-headed sperm syndrome, is characterized by a large number of acrosome absences, nuclear membrane abnormalities, and midsection defects in the ejaculatory ducts. Mutations or deletions in SPATA16, PICK1 and DPY19L2 can cause sperm DNA damage and subsequently cause globozoospermia when sperm repair is abnormal. For example, Houda et al. found that when complete globozoospermia lacks exon 2 of SPATA16 gene, it will interfere with protein synthesis and meiosis in the process of sperm formation, which may not only lead to globozoospermia, but also lead to the appearance of other malformations such as multiple tails, multiple heads, and double heads of sperm (155). In addition, Koscinski et al. performed gene analysis on four brothers with globozoospermia and found homozygous deletion of a gene fragment with a length of 200 kb on chromosome 12, and this fragment only covered the DPY19L2 gene (156).

##### 3.2.1.2 Oligozoospermia or azoospermia

**Figure 4:**
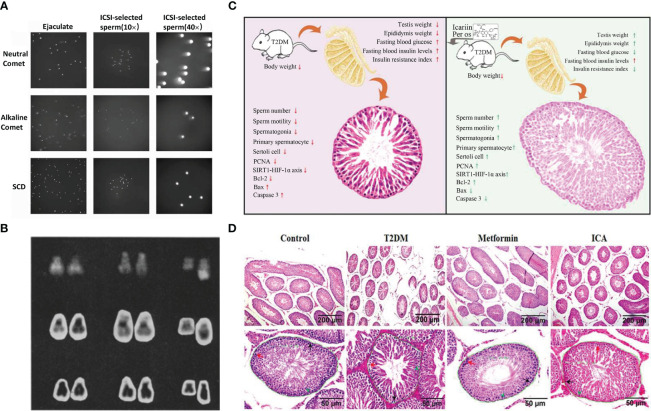
Comet analysis of spermatozoa, contrast of chromosomes between anspermatic and normal males, ICA protection against diabetes-induced testicular dysfunction in rats and schematic diagram of testicular tissue staining. **(A)** Microscopic images of ejaculated and ICSI-selected spermatozoa analyzed by Neutral Comet, Alkaline Comet, SCD (159). **(B)** From left to right: chromosome pairs 21 and 22 and deleted g from 3 of azoospermie men. Last chromosome in each row is normal paternal Y. *From top*, Q hands, C bands, and orcein (158). **(C)** Schematic illustrating ICA’s protection against diabetes mellitus induced testiculardysfunction in rats possibly via enhancing cell proliferation and inhibiting intrinsic mitochondria dependent apoptotic signaling. Red arrows in T2DM panel mean deleterious effects (with significant difference) to reproductive function and relative parameters when compared with normal control group (nondiabetic). Red and green arrows in ICA treatment panel represent that there are no and obvious effectiveness respectively after ICA administration when compared with T2DM group (160). **(D)** Hematoxylin and eosin staining of testicular tissue. The lower panes show partial amplifcation of upper panes. In each seminiferous tubule, the black arrow points to a representative spermatogonia, the red arrow, primary spermatocyte, and the green arrowhead, Sertoli cell. In addition, the green curves present regions used to analyze the cross-sectional area of seminiferous tubule whose value displayed beside (160).

In recent years, more and more studies have found that sperm production is regulated by many genes, and the mutation, abnormal expression or deletion of these genes may change the male reproductive ability, leading to spermatogenesis disorders and oligospermia or azoospermia. Relevant studies have shown that microdeletions have been found in three non-overlapping regions of AZF a-b-c (144) on the Y chromosome of males, including RBM39, DAZ, DFFRY40, DBY and CDY. If these lesions are not repaired in time or the repair is abnormal, it may lead to oligospermia or azoospermia. For example, complete azoospermia occurs when two genes, DFFRY and DBY, are deleted together (157). In addition, Tiepolo et al. found that 6 azoospermia patients had a deletion of the long arm of the Y chromosome, and therefore, It is hypothesized that there is a gene controlling spermatogenesis in the nonfluorescent region of the long arm of the Y chromosome (Yq11.23), which is named “azoospermia factor” because this abnormality is present in many men with azoospermia (158) (**Figure 4B**).

##### 3.2.1.3 Asthenozoospermia

Sperm nuclear DNA damage is one of the important mechanisms leading to asthenozoospermia. The respiratory and metabolic functions of mitochondria in severely damaged sperm will be significantly reduced, and sperm motility will also be weakened. The decrease of mitochondrial respiratory function is closely related to the damage of sperm nucleus. Autophagy is the process by which cells break down some harmful parts of themselves and recycle them. Studies have shown that the repair mechanism of autophagy in sperm can remove the damage from mitochondrial DNA, thereby repairing the damaged mitochondrial DNA. At the same time, REDOX mechanisms may also be a mode of mitochondrial DNA repair. Studies have shown that spermatozoa have the ability to repair oxygen free radical damage under oxidative stress. This ability is achieved through REDOX mechanisms. In addition, mitochondrial RNA is a small RNA molecule that can alleviate mitochondrial DNA damage by breaking down the damaged mitochondrial RNA, which is also involved in the process of sperm repair of mitochondrial DNA damage. Increasing the content of mitochondria in sperm or improving the function of mitochondria can inhibit sperm DNA damage to a certain extent, thereby reducing sperm apoptosis and DNA damage and improving sperm quality. Mitochondria in the middle of sperm are the energy generators of mammalian sperm, and sperm need mitochondrial oxidative phosphorylation to provide sufficient adenosine triphosphate for normal function (147). During spermatogenesis, sperm mitochondria undergo drastic morphological changes and subcellular reorganization. In order to maintain an appropriate number of mitochondria in sperm, the replication and subcellular organization of mitochondrial DNA must be accurately controlled during spermatogenesis, otherwise it may cause asthenospermia. For example, Kao et al. found that the relative content of mtDNA in the sperm of men with asthenospermia was significantly lower than that of normal men, and the human sperm with low motility score had multiple deletions of mtDNA (161), so they speculated that the deletion of mitochondrial DNA would lead to asthenospermia.

#### 3.2.2 Ovum

During foetal ovarian development, a large number of germ cells undergo apoptosis; however, the cause remains unclear. There are several hypotheses for this, but it is generally accepted that defective ovum death and germ cell apoptosis may be related to the quality of germ cell DNA, mainly due to a reduction in DNA integrity. The reduction in foetal DNA integrity may be related to ROS in germ cells, and the ROS content is also related to the nutritional status of the mother. Some relevant data show that in the early stages before meiotic arrest and primordial follicle assembly, the sterility of germ cells could be caused by abnormal DNA damage repair and homologous recombination. The repair mode of egg cell itself mainly includes mismatch repair and base excision repair. By initiating specific molecular pathways to identify and locate the damage site, the corresponding repair mechanism is initiated, and finally the repair of DNA damage is realized.

##### 3.2.2.1 Polycystic ovary syndrome

Polycystic ovarian syndrome (PCOS) is the most common reproductive endocrine and metabolic disorder worldwide (162). Abnormal follicular development and poor oocyte and embryo quality can lead to abnormal ovulation and polycystic ovary syndrome. Long-term metabolic disorders also have adverse effects in patients with PCOS (163, 164). Increased secretion and content of ovarian steroids in PCOS will produce OS, which leads to DNA damage. The process of DNA damage repair in egg cells mainly includes nucleotide excision repair, homologous recombination repair, etc. In nucleotide excision repair, if there are some mispaired bases or small defects in the DNA strand, the cell will first call the corresponding enzyme to cut the wound, and then the polymerase and other enzymes will add new nucleotides to the incision, so that the DNA strand can be completely connected. In homologous recombination repair, cells can repair DNA damage by using the same sequence region on another homologous chromosome, which ensures the accuracy of the DNA sequence and the wide range of variability. If not repaired in time or the repair process is abnormal, germ cell development may be arrested or apoptosis in germ cells may be triggered. For example, Chen et al. found that abdominal obesity can aggravate oxidative damage in the adipose tissue of patients with PCOS, and that OS can cause cell damage, leading to dysfunction of adipocytes in cytokine secretion, fat accumulation, and obesity (165).

Oxidative metabolism is an important intraovarian regulatory pathway in the follicles. The ovary produces a dominant follicle approximately every month that controls the increase in ROS production during this process. At the same time, it is inhibited by antioxidants, which can inhibit the DNA damage caused by OS (166). The occurrence of PCOS is largely related to a decrease in antioxidant concentration. Free radicals and antioxidants play crucial roles in maintaining the ovarian environment during oocyte development and the luteal phase. In related studies, patients with PCOS were found to be 1.7 times more likely to develop endometrial cancer than healthy women, which has much to do with OS due to PCOS. In addition, OS is also involved in the mechanism of unexplained infertility, endometriosis, male infertility, and related impaired ovum quality (167). Chronic accumulation of ROS can also lead to tissue damage in the reproductive system (149). PCOS can also lead to related complications and also cause the balance between antioxidant mechanisms and ROS to be affected, resulting in an increase in ROS, which in turn destroys cell membrane lipids and leads to lipid peroxidation (168). Uncertain factors such as endocrine disorders may also affect the ability of cells to perform DNA damage repair, making the incidence of DNA damage in germ cells increased. Therefore, in the treatment of PCOS, it is necessary to control endocrine disorders, avoid damage to germ cell DNA by harmful factors as much as possible, and assist patients to repair germ cell DNA damage.

##### 3.2.2.2 Primary ovarian insufficiency

Primary Ovarian Insufficiency (POI) is a common disease in gynaecological endocrinology, and its clinical manifestations include delayed puberty, primary amenorrhoea (PA), and secondary amenorrhoea (SA) (169). POI is usually caused by chromosomal abnormalities in germ cells, such as chromosome breaks, crossotypes, or chromosome deletions. These abnormalities cause DNA damage, which in turn leads to the activation of DNA repair systems in germ cells. Changes in DNA damage repair genes may lead to chromosomal breakage, cytopathic effects, and the development of cancer, which increases the risk of POI. The repair of DNA damage mainly includes direct repair, indirect repair and recombination repair. In the direct repair process, the repair methods include base excision repair and photodamage repair. At the same time, in the process of indirect repair, germ cells will fill the damaged region through repeat pairing of DNA, such as non-homologous end joining repair and homologous recombination repair. In addition, germ cells will repair the damage through homologous recombination, including unilateral gene repair and bilateral gene repair. In addition, members of the Fanconi anaemia complementation group (FANC) have defects in DNA repair after replication, which leads to decreased fertility and primary ovarian insufficiency. For example, Yang et al. detected two rare heterozygous mutations in FANCA in sporadic POI cases (170).

POI is primarily caused by genetic, single-factor, or chromosomal abnormalities. Primary ovarian dysgenesis can be caused by autoimmune diseases, viral infections, and/or toxins (169, 171). In premeiotic stage I, the establishment of an association complex (SC) and the repair of DNA double-strand breaks are required. The correct formation of SC requires the aid of a ring of cohesion around the chromosome, such as recombinant 8 (REC8) encoding, matrix antigen 3 (STAG3) encoding, radiation-sensitive 21-like (RAD21L) encoding, and other coproteins, which are specific for meiosis. In some patients with familial POI, the deletion of these proteins may lead to oocyte apoptosis or mutations during meiosis (151, 152). For example, Pu et al. performed whole-exome sequencing analysis in a Chinese nuclear family with POI, and compound heterozygous mutation of HFM1 gene was found in the genome of one pair of sisters with POI. Subsequently, another patient with a compound heterozygous mutation of HFM1 was found in a small sample of patients with sporadic POI, and the close relationship between HFM1 mutation (9/138) and POI was further confirmed in a study with an expanded sample size (172).

## 4 Therapies

Although attention to DNA damage in reproductive diseases is gradually increasing, there are relatively few clinical treatment methods for such diseases. Currently, drugs commonly used to treat DNA damage and reproductive diseases include icariin (173), antioxidants (174), and poly (ADP-ribose) polymerase (PARP) inhibitors (175). All these are repaired at the DNA level, and commonly used treatment methods include intracytoplasmic sperm injection (ICSI) (176, 177) and varicocele surgery.

### 4.1 Drug therapy

Drugs for the treatment of reproductive diseases caused by DNA damage have been used in both traditional Chinese and Western medicine; however, their development is still at an immature stage. Drug therapy is a conservative treatment unlikely to be successful. Most drugs play only a regulatory role, but their risk is low, and they are of great significance in the field of DNA damage. Traditional treatment drugs mainly include icariin, and new treatment drugs include antioxidants and poly (ADP-ribose) polymerase inhibitors.

#### 4.1.1 Icariin

Icariin (ICA), the main active ingredient of Epimedium, is a flavonoid traditionally used to treat erectile dysfunction (178). ICA can reduce DNA damage in testicular germ cells and ameliorate the decline in reproductive function caused by sperm DNA damage. Moderate doses of ICA can promote spermatogenesis by increasing the expression of steroidogenic acute regulatory protein (StAR) and peripheral benzodiazepine receptor (PBR). This increases the level of follicle-stimulating hormone receptor (FSHR) and subsequent increase in testosterone secretion, which is an indispensable element in the steroidogenic mechanism that synergistically transfers cholesterol into the mitochondria (148). For example, Chen et al. found that icariin participates in testosterone production by regulating the mRNA expression of StARs and peripheral benzodiazepine receptors (PBR). Moreover, the content of icariin in 50 mg·kg^-1^·d^-1^ or 100 mg· kg^-1^·d^-1^ could significantly increase the testosterone level of rats (179). In addition, Nan et al. found that ICA stimulates the proliferation of Sertoli cells, which play an important role in regulating the self-renewal and differentiation of spermatogonial stem cells (180). For example, Zhou et al. found that ICA could inhibit, *in vitro* and *in vivo*, the Akt pathway through the blood-testis barrier, thereby inhibiting damage to gap junction communication and the gap junction protein connexin 43 caused by microcystin (LR subtype). Thus, the damage caused by microcystins (LR subtype) in Sertoli cells was alleviated (143). Studies show that ICA could effectively reduce the male reproductive dysfunction and spermatogenesis defects induced by diabetes mellitus (DM), promote the release of testosterone, inhibit the expression of testicular type IV collagenase and transforming growth factor-β-1, and thus protect the testicular damage caused by diabetes mellitus. In addition, ICA can improve DM-induced spermatogenesis defects through the proliferation of spermatogonia, primary spermatocytes, and Sertoli cells and the inhibition of mitochondria-dependent apoptotic pathways (**Figure 4C**). Therefore, ICA can be used as a potential new therapeutic agent for the protection and treatment of DM-induced testicular damage (160) (**Figure 4D**).

#### 4.1.2 Antioxidant

One of the causes of sperm DNA damage is ROS generation, which can be prevented using antioxidants (150). According to their biochemical classification, antioxidants are divided into two main categories: enzymatic and non-enzymatic. Enzymatic antioxidants are key natural enzymes that can remove excess ROS and reactive RNS. Superoxide dismutase (SOD), catalase, and glutathione peroxidase are the primary antioxidant enzymes. Non-enzymatic antioxidants are compounds with low molecular masses such as glutathione, ascorbic acid, and flavonoids. Some studies have reported that ascorbic acid (600 mmol/L),α-tocopherol (30 and 60 mmol/l), and uric acid (400 mmol/l) have significant protective effects against sperm DNA damage induced by X-ray irradiation *in vitro* (P < 0.001). Therefore, *in vitro* supplementation with these antioxidants alone may have beneficial effects on sperm DNA integrity. In addition, isoflavones, which are plant compounds including genistein and equol, have a potential positive role in the prevention of male infertility owing to their physiological effects on antioxidant activity. Genistein showed the strongest antioxidant effect at physiological concentrations, followed by equol, ascorbic acid, and α-tocopherol. When genistein and equol were used together, the protective effect was stronger than that when they were used alone, thereby preventing sperm DNA damage (100). For example, Scaruffi et al. evaluated the reproductive outcomes of IVF cycles after treatment with 2 Gametogen^®^ tablets. The tablet contains a variety of antioxidants including alpha-lipoic acid (800 mg), folic acid (400 mg), coenzyme Q10 (200 mg), inositol (1000 mg), vitamins B2 (2.8 mg), B6 (2.8 mg), and B12 (5µg), zinc (15 mg), and selenium (83µg). The results showed significant increases in sperm motility and pregnancy rates from 3% before treatment to 33% at 12 weeks after treatment (181).

#### 4.1.3 Poly (ADP-ribose) polymerase inhibitors

The mechanism of action of poly (ADP-ribose) polymerase (PARP) inhibitors is their inhibition of the repair process of DNA single-strand damage. However, this type of single-strand DNA damage can be converted into double-strand damage (DSB) during DNA replication to form replication forks, and DSB can be repaired by homologous recombination (HR). Related studies have found that the combination of PARP and other targeted drugs for the treatment of infertility caused by DNA damage will be of great interest in the next few years. Moreover, poly (ADP-ribose) polymerase inhibitors have led to breakthroughs in ovarian cancer clinical trials. Compared to ovarian cancer, research on PARP inhibitors in uterine malignant tumors is still in its infancy; however, current clinical studies have shown that PARP inhibitors have a certain application potential. The combination of PARP inhibitors with targeted drugs such as ICIs, angiogenesis inhibitors, and tyrosine kinase inhibitors is expected to have positive remedial effects on patients. In addition, PARP inhibitors have been used to treat ovarian cancer and have shown significant effects (182). For example, Cheng et al. conducted a search of relevant clinical trial registries for randomized controlled trials comparing PARP inhibitors and placebo in women with newly diagnosed epithelial ovarian cancer. They found a significant clinical benefit of PARP inhibitors in progression-free survival (PFS) compared to placebo in maintenance therapy with homologous recombination deficiency (HRD). Therefore, maintenance therapy with PARP inhibitors can significantly prolong PFS in patients with newly diagnosed ovarian cancer (183).

#### 4.1.4 Nanomedicine

Nanomaterials refer to natural or man-made objects (184) with at least one nanometer size (1-100 nm) in three dimensions, including quantum dots (QD), upconversion nanomaterials, and two-photon absorption nanomaterials. By specifically accumulating in tumors, nanomedicine can ensure the delivery and release of tumor-targeted drugs through passive and active targeting methods, which helps to maintain the balance between efficacy and toxicity. Nanomedicine has the advantages of increasing the solubility of chemotherapy drugs, encapsulating multiple drugs and improving biodistribution. The core of nanomedicine is the nanoscale technology of drugs, including direct nanoscale drug delivery and nano-drug delivery system. The application of nanotechnology in the treatment of reproductive diseases is currently in its infancy. The possibilities of organic and inorganic nanomaterials in reproductive treatment mainly include targeted labeling of sperm populations for sorting purposes (185) and experimental gene therapy for monogenic disorders in fetuses *in utero*, bioimaging (159), and labeling of preimplantation embryos. To facilitate their tracking during *in vitro* fertilization (IVF) culture (186). At the same time, nanomaterials have been used to deliver compounds to sperm to assess their functional impact on intracellular pathways (187) and to promote sperm-mediated gene transfer (SMGT) based on the intrinsic ability of sperm to spontaneously bind and integrate exogenous DNA (188). For example, Dai et al. (189) studied human prostate cancer cell lines with respect to carbon nanotubes (CNTS) and confirmed the use of CNTS as novel site-directed compounds for targeting prostate cancer (targeted drug delivery). In addition, the use of nanoparticles (NPS) for the diagnosis and treatment of common diseases affecting many women of reproductive age, such as endometriosis and uterine fibroids, is in progress, and there is increasing evidence that the use of NPS can accurately diagnose cancer at an early stage and facilitate effective cancer treatment in women. Coupling of ligands targeting cancer-specific receptors with paramagnetic NPS became the mainstay of treatment. For example, Yang et al. showed that ferroparamagnetic NP was coated with a poly (amino acid) derivative (PAION Ab) 121 conjugated to the tyrosine kinase human epidermal growth factor receptor 2 (HER2)/neu antibody and showed specific targeting to SKBR-3 cells, which overexpressed the receptor. Therefore, it has the potential to become a good new magnetic resonance (MR) contrast agent (190).

#### 4.1.5 Small molecule inhibitors

Small molecule inhibitors generally refer to enzyme inhibitors, which are a class of organic compound molecules that can target proteins, hinder biochemical reactions or reduce protein activity. Their molecular weight is generally less than 1000 Dalton and has cell permeability. In addition, most of the drugs are used clinically, and their main components are small molecule inhibitors. The target proteins of small molecule inhibitors are generally enzymes, and small molecules can directly bind to these target proteins, thereby reducing protein activity by competing with substrates, changing protein structure, or hindering protein conformational transition. At present, it is mainly used to treat cancer, especially those caused by germ cells carcinogenesis, and small molecule inhibitors are highly selective and cell permeable. c-Met is a receptor tyrosine kinase with a high affinity ligand hepatocyte growth factor/dispersion factor (HGF/SF). The dysregulation of hepatocyte growth factor (HGF) receptor c-Met contributes to tumor progression and metastasis. Therefore, inhibition of this pathway will be an effective therapeutic approach for the treatment of tumors. For example, the aim of the Marion et al. study was to determine whether blockade of c-Met by the oral c-Met inhibitor PF-2341066 would improve the productivity of a xenograft model of ovarian cancer metastasis as well as reduce tumor burden. Studies have shown that this inhibitor only reduces tumor growth and metastasis by about 60%, and HGF-c-Met signaling is only one of the oncogenic signaling pathways that promote tumorigenesis. Therefore, combining c-Met inhibitors with other targeted therapies or chemotherapy may be effective for the treatment of ovarian cancer or other cancers (191). In addition, Jin et al. found that API-59CJ-Ome inhibited the AKT pathway and subsequently induced apoptosis in highly expressed endometrial cancer cell lines (192).

### 4.2 Operative treatment

Currently, there are few studies on the surgical treatment of DNA damage in reproductive diseases, and these are still in the developmental stage. Although the risk of surgical treatment is high, it has the advantages of less time consumption and a high success rate of pregnancy for the treatment of DNA damage and reproductive diseases and plays an important role in the field of DNA therapy. Surgical treatments can be divided into two categories. The first is to use assisted reproductive technology, such as intracytoplasmic sperm injection (ICSI), to successfully fertilize the ovum and sperm, and the second is to fundamentally reduce the DNA damage rate, such as varicocele surgery.

#### 4.2.1 Intracytoplasmic sperm injection

Intracytoplasmic sperm injection (ICSI), in which a woman who is not yet pregnant is conceived by means of *in vitro* fertilization (IVF) or oocyte subzone fertilization (SUZI), offers the most reliable and consistent chance of fertilization because it bypasses all the initial steps of cumulus cell penetration, zona pellucida bonding, and sperm fusion with the oocyte during natural fertilization. Relevant studies have shown that many reproductive abnormalities are related to sperm DNA damage, including impaired fertilization, interrupted pre-implantation embryo development, abortion, and offspring birth defects (153). Currently, ICSI is widely used in assisted reproduction and provides relevant information about the basic science of fertilization. Sperm DNA fragmentation (SDF), measured by sperm chromatin dispersion, has an important impact on morphological dynamic parameters, fertilization rate, and blastocyst quality in ICSI treatment. For example, Wang et al. found that 15% SDF reduced fertilization rates in ICSI cycles and affected morphodynamic parameters. Higher SDF levels can also induce higher cleavage rates, resulting in lower blastocyst formation rates and blastocyst quality (193). Lara-Cerrillo et al. found that sperm selection during ICSI treatment resulted in a large decrease in single-stranded DNA break values (194) (**Figure 4A**).

#### 4.2.2 Varicocele surgery

Varicocele is a vascular disease characterized by the regurgitation of blood from the spermatic vein due to its obstruction or failure of valve function, resulting in abnormal expansion, elongation, and tortuosity of the pampiniform plexus. It is a common clinical condition that leads to male infertility (145, 146). Varicoceles occur in approximately 15%–20% of the general male population and are the most common cause of decreased semen quality. Studies have shown that DNA damage and sperm nuclear abnormalities are significantly higher in men with varicocele than in healthy men. Treatment of this disease mainly includes conservative and surgical approaches. Varicocele surgery is widely used to treat male infertility and can significantly reduce DNA damage. For example, Pourmand et al. used the triphosphate nick-end labeling (TUNL) method to analyse semen and assess sperm DNA damage in patients with varicocele, and the results showed that DNA damage significantly improved after varicocelectomy. This indicates that varicocele surgery has an important application value in the treatment of DNA damage in reproductive diseases (195).

#### 4.2.3 Gene therapy

Gene therapy is a therapeutic technology that interferes with the physiological function of cells at the nucleic acid level. It can treat diseases caused by gene abnormalities by introducing normal genes to replace missing or abnormal mutated genes, or inhibiting the function of abnormal endogenous genes. The target cells in gene therapy are mainly divided into germline cell therapy and somatic cell therapy. The former can transmit the introduced genes to the offspring, while the latter, on the contrary, is mainly germline cell therapy for infertility. One of the main focuses of this technology is the optimization of plasmids, nanostructures, or viral delivery vectors. Due to the outstanding performance of virus in cell invasion and insertion of genetic material, it is a more mainstream research direction. In addition, it may be very difficult to perform gene therapy on testis and sperm without affecting the germline, so the use of sperm-mediated gene transfer and testis-mediated transgene as methods to generate transgenic animals will be of great interest for future research. “For example, Nagano et al. showed that retroviral vectors can deliver transgenes into spermatogonial stem cells *in vitro* and showed that transfer of such transgenic stem cells into the testes of mice in which their own spermatogonial stem cells had been depleted resulted in repopulation of testes and donor cells.” The transgene is subsequently integrated into the chromosomes of the offspring (196).

#### 4.2.4 Stem cell therapy

Infertility is a globally recognized problem caused by a variety of reproductive diseases. To date, many treatments have been tried to restore fertility, and although some have yielded results, it is not possible to restore fertility in all cases. Stem cells (SCs) have the ability to self-renew, differentiate into undifferentiated cells of multiple cell types and produce different paracrine factors for regulation. Therefore, the use of stem cell therapy holds great promise for the treatment of infertility patients. The treatment of non-obstructive azoospermia is a difficult problem in the field of reproductive medicine, and it is also an important cause of male infertility. Spermatogonial stem cells are the basis of male spermatogenesis and fertility. The most common causes of non-obstructive azoospermia are chromosome karyotype abnormality, Y chromosome microdeletion and gene mutation, and the treatment of spermatogonial stem cells plays an important role in spermatogenesis and fertility. For example, Rahbar et al. found that in three dimensional culture system with a small body of epididymis cocultivation of spermatogonial stem cells can effectively improve sperm without mouse spermatogenesis process (197). In addition, many studies have shown that MSCS have the ability to restore function for the treatment of premature ovarian failure diseases. For example, Huang et al. found that human amniotic fluid mesenchymal stem cells can resist DNA damage and ameliorate premature ovarian failure (198).

## 5 Conclusion and prospect

This review summarizes recent advances in understanding the role and mechanism of DNA damage repair in germ cell development. First, we introduce the mechanisms, repair methods, and applications of DNA damage repair. Second, we summarized the causes of DNA damage and detection methods of DNA damage, among which the detection methods for DNA damage were different as well as their principles of action. Third, we summarized the causes of abnormal DNA repair in germ cells and their effects on sperm and ovarian cells, provided some common examples, and summarized drugs and treatments for reproductive diseases, including icariin, antioxidants, poly (adenosine diphosphate-ribose) polymerase (PARP) inhibitors, intracytoplasmic sperm injection (ICSI), and varicocelectomy. Finally, we summarized the relevant treatments drug and treatments methods for reproductive diseases, including icariin, antioxidants, and polyadenosine ribosylphospho (PARP) inhibitors, as well as treatments such as intracytoplasmic sperm injection (ICSI) and varicocele surgery.

In conclusion, DNA damage and DNA damage repair are closely related to the occurrence and treatment of a variety of reproductive diseases caused by DNA damage. Research on the mechanism of DNA damage repair in the treatment of reproductive diseases can provide a deeper understanding of the mechanism of reproductive disease treatment. This provides ideas that are of great significance for the development and exploration of therapeutic targets for diseases caused by abnormal DNA damage and repair. At present, research and treatment methods for reproductive diseases caused by DNA damage or abnormal DNA damage repair in germ cells are increasingly mature; however, some known theories have not been validated, some treatment methods have unknown areas, and few treatment methods can be widely used in clinical practice. Therefore, further research should focus on elucidating the specific mechanisms that cause sperm DNA damage and the mechanism of DNA damage repair, so as to provide a basis for the treatment of sperm DNA damage that has been caused.For example, At present, the research on the mechanism of sperm DNA integrity mainly focuses on oxidative stress response, apoptosis and chromatin abnormality, but the specific mechanism has not been fully elucidated. The study of sperm DNA integrity is of great theoretical and clinical value in andrology. The related factors, mechanism and traditional Chinese medicine treatment of sperm DNA integrity need to be further studied. At the same time, the existing detection technology of sperm DNA integrity is still unable to meet the needs of clinical and scientific research, so finding an ideal detection method for the diagnosis and treatment of male infertility is also the trend of future research. In addition, much less is known about the mechanisms of DNA damage repair in female germ cells than in males. Maternal obesity can cause chronic ovarian inflammation and decreased oocyte quality. However, the mechanism of maternal obesity damage to embryonic development and offspring health is still questionable, and strategies to improve its adverse reproductive outcomes also need to be further studied. It is hoped that further research will solve and understand the current problems of DNA damage repair in germ cells. With the development of medicine and biotechnology, DNA damage and damage repair abnormalities in germ cells can be studied further, and their treatment methods can be improved and popularized in clinical applications to effectively improve human life.

## Author contributions

Detailed author contributions: QL and LL conceptualized and planned the manuscript. YW and MS wrote the original draft of the paper. YC reviewed the original manuscript. XH and LR performed the literature search. All authors contributed to the article and approved the submitted version.

## References

[1] Beatriz F Max MI Tinna S. Measuring the activity of DNA repair enzymes in isolated mitochondria. Methods Mol Biol (2022) 2363:321–34. doi: 10.1007/978-1-0716-1653-6_21 34545501

[2] Kara A Özgür A Nalbantoğlu S Karadağ A. DNA Repair pathways and their roles in drug resistance for lung adenocarcinoma. Mol Biol Rep (2021) 48(4):3813–25. doi: 10.1007/S11033-021-06314-Z 33856604

[3] Walker GC Nimrat C. Mechanisms of DNA damage, repair, and mutagenesis. Environ Mol Mutagen (2017) 58(5):235–63. doi: 10.1002/em.22087 PMC5474181 28485537

[4] Carusillo A Mussolino C. DNA Damage: from threat to treatment. Cells (2020) 9(7):1665. doi: 10.3390/cells9071665 32664329 PMC7408370

[5] Iyer RR Pluciennik A Burdett V Modrich PL. DNA Mismatch repair: functions and mechanisms. Chem Rev (2006) 106(2):302–23. doi: 10.1002/chin.200620268 16464007

[6] Loon BV Markkanen E Hübscher U. Oxygen as a friend and enemy: how to combat the mutational potential of 8-oxo-guanine. DNA Repair (2010) 9(6):604–16. doi: 10.1016/j.dnarep.2010.03.004 20399712

[7] Sriram K Joseph J Schuster GB Barnett RN Cleveland CL Landman U. Oxidation of DNA: damage to nucleobases. Accounts Chem Res (2010) 43(2):280–7. doi: 10.1021/ar900175a 19938827

[8] Chakarov S Petkova R Russev GC Zhelev N. DNA Damage and mutation. types of DNA damage. Biodiscovery (2014) 11(11):1. doi: 10.7750/BioDiscovery.2014.11.1

[9] Cai Z Fu M Liu K Duan X. Therapeutic effect of Keap1-Nrf2-ARE pathway-related drugs on age-related eye diseases through anti-oxidative stress. Int J Ophthalmol-Chi (2021) 14(8):1260–73. doi: 10.18240/IJO.2021.08.19 PMC8342288 34414093

[10] Kurt H JJ S Tuhin H Md ASB Tanhaul I Amanda W. Formation and repair of unavoidable, endogenous interstrand cross-links in cellular DNA. DNA Repair (2020) 98:103029. doi: 10.1016/J.DNAREP.2020.103029 33385969 PMC8882318

[11] Park VS Pursell ZF. POLE proofreading defects: contributions to mutagenesis and cancer. DNA Repair (2019) 76:50–9. doi: 10.1016/j.dnarep.2019.02.007 PMC6467506 30818169

[12] Karahan G Chan D Shirane K McClatchie T Janssen S Baltz JM. Paternal MTHFR deficiency leads to hypomethylation of young retrotransposons and reproductive decline across two successive generations. Development (2021) 148(13):dev199492. doi: 10.1242/DEV.199492 34128976 PMC8276981

[13] Vidal AC Henry NM Murphy SK Oneko O Nye M Bartlett JA. PEG1/MEST and IGF2 DNA methylation in CIN and in cervical cancer. Clin Transl Oncol (2014) 16(3):266–72. doi: 10.1007/s12094-013-1067-4 PMC3924020 23775149

[14] Qing L Yuanyuan L Qi Y Shuo H Kai W Liangchai Z. Temporal regulation of prenatal embryonic development by paternal imprinted loci. Sci China Life Sci (2020) 63(1):1–17. doi: 10.1007/s11427-019-9817-6 31564034

[15] Cadet J Douki T Gasparutto D Ravanat J-L. Oxidative damage to DNA: formation, measurement and biochemical features. Mutat Res-Fund Mol M (2003) 531(1):5–23. doi: 10.1016/j.mrfmmm.2003.09.001 14637244

[16] Griveau JF Lannou DL. Reactive oxygen species and human spermatozoa: physiology and pathology. Int J Androl (1997) 20(2):61–9. doi: 10.1046/j.1365-2605.1997.00044.x 9292315

[17] Tran LV Malla BA Kumar S Tyagi AK. Polyunsaturated fatty acids in Male ruminant reproduction [[/amp]]mdash; a review. Asian Austral J Anim (2017) 30(5):622–37. doi: 10.5713/ajas.15.1034 PMC5411821 26954196

[18] Takeshima T Kuroda S Yumura Y. Reactive oxygen species and sperm cells. ROS Living Cells (2018). doi: 10.5772/intechopen.73037

[19] Shuang Z Shan J Min W Chunyan C Xin W. A theoretical study towards understanding the origin of DNA oxidation products. J Phys Org Chem (2020) 34(5). doi: 10.1002/POC.4176

[20] Pengfei Y Xiangxia L Jin L Tianyi Z Xiaoling G Junrui H. Ionizing radiation upregulates glutamine metabolism and induces cell death via accumulation of reactive oxygen species. Oxid Med Cell Longev (2021) 2021:5826932. doi: 10.1155/2021/5826932 35028001 PMC8749225

[21] Robert N Ang LN. Nuclear DNA damages generated by reactive oxygen molecules (ROS) under oxidative stress and their relevance to human cancers, including ionizing radiation-induced neoplasia part II: relation between ROS-induced DNA damages and human cancer. Radiat Med Prot (2020) 1(3):140–52. doi: 10.1016/J.RADMP.2020.11.003

[22] Yeliz Y Özel Tİ Seda TAR. Ionizing radiation induced DNA damage via ROS production in nano ozonized oil treated B-16 melanoma and OV-90 ovarian cells. Biochem Bioph Res Co (2022) 615:143–9. doi: 10.1016/J.BBRC.2022.05.030 35623299

[23] Adong K Dan X Tingting H Qiangde L Rui Z Kangsen M. Role of acyl-coenzyme a oxidase 1 (ACOX1) on palmitate-induced inflammation and ROS production of macrophages in large yellow croaker (Larimichthys crocea). Dev Comp Immunol (2022) 136:104501. doi: 10.1016/J.DCI.2022.104501 35961593

[24] Prahlad Maremanda K Khan S Jena GB. Role of zinc supplementation in testicular and epididymal damages in diabetic rat: involvement of Nrf2, SOD1, and GPX5. Biol Trace Elem Res (2016) 173(2):452–64. doi: 10.1007/s12011-016-0674-7 27025721

[25] Shokolenko IN Wilson GL Alexeyev MF. Aging: a mitochondrial DNA perspective, critical analysis and an update. World J Exp Med (2014) 4(4):46–57. doi: 10.5493/wjem.v4.i4.46 25414817 PMC4237642

[26] Aitken RJ Smith TB Jobling MS Baker MA Iuliis GND. Oxidative stress and male reproductive health. Asian J Androl (2014) 16(1):31–8. doi: 10.4103/1008-682X.122203 PMC3901879 24369131

[27] Nabil A SR A SR K Iwan L-J Navid E TA J. Novel association between sperm reactive oxygen species production, sperm morphological defects, and the sperm deformity index. Fertil Steril (2004) 81(2):349–54. doi: 10.1016/j.fertnstert.2003.06.026 14967372

[28] Koppers AJ Garg ML Aitken RJ. Stimulation of mitochondrial reactive oxygen species production by unesterified, unsaturated fatty acids in defective human spermatozoa. Free Radical Bio Med (2009) 48(1):112–9. doi: 10.1016/j.freeradbiomed.2009.10.033 19837155

[29] Furmanchuk A Isayev O Gorb L Shishkin OV Hovorun DM Leszczynski J. Novel view on the mechanism of water-assisted proton transfer in the DNA bases: bulk water hydration. Phys Chem Chem Phys (2011) 13(10):4311–7. doi: 10.1039/c0cp02177f 21253641

[30] Sambrano JR Souza AR Queralt JJ Andrés J. A theoretical study on cytosine tautomers in aqueous media by using continuum models. Chem Phys Lett (2000) 317(3-5):437–43. doi: 10.1016/S0009-2614(99)01394-9

[31] Jarrett SG Wolf Horrell EM Christian PA Vanover JC Boulanger MC Zou Y. Retraction notice to: PKA-mediated phosphorylation of ATR promotes recruitment of XPA to UV-induced DNA damage. Mol Cell (2022) 82(22):4400. doi: 10.1016/J.MOLCEL.2022.10.023 36400011 PMC9721266

[32] Paganini DA Lummertz MM Salvan DL Luz BÂCd Jacobsen SR Martins LL. Royal jelly reduce DNA damage induced by alkylating agent in mice. Mutat Res (2022) 825:111796. doi: 10.1016/J.MRFMMM.2022.111796 36007462

[33] Ying Y Jun D Fa Z Xiao L. Vitamin e and selenium partially prevent cytotoxicity, oxidative stress and DNA damage induced by T-2 toxin in bovine leydig cells. Theriogenology (2022) 189:255–61. doi: 10.1016/J.THERIOGENOLOGY.2022.06.028 35809359

[34] Hong S. DNA Damage response is hijacked by human papillomaviruses to complete their life cycle. J Zhejiang Univ-Sc B (2017) 18(3):215–32. doi: 10.1631/jzus.B1600306 PMC5369246 28271657

[35] Pfeifer G You YH Besaratinia A. Mutations induced by ultraviolet light. Mutat Res (2005) 571(1-2):19–31. doi: 10.1016/j.mrfmmm.2004.06.057 15748635

[36] Gates KS. An overview of chemical processes that damage cellular DNA: spontaneous hydrolysis, alkylation, and reactions with radicals. Chem Res Toxicol (2009) 22(11):1747–60. doi: 10.1021/tx900242k PMC2806061 19757819

[37] Zhang M QA A GA C Lindsay F J Jiali H. Use of tanning beds and incidence of skin cancer. J Clin Oncol (2012) 30(14):1588–93. doi: 10.1200/JCO.2011.39.3652 PMC3383111 22370316

[38] Baulch JE Li M Raabe OG. Effect of ATM heterozygosity on heritable DNA damage in mice following paternal F0 germline irradiation. Mutat Res (2007) 616(1-2):34–45. doi: 10.1016/j.mrfmmm.2006.11.020 17161850

[39] Tateno H Kusakabe H Kamiguchi Y. Structural chromosomal aberrations, aneuploidy, and mosaicism in early cleavage mouse embryos derived from spermatozoa exposed to gamma-rays. Int J Radiat Biol (2011) 87(3):320–9. doi: 10.3109/09553002.2011.530334 21087169

[40] Said RS Nada AS Ebtehal ED Pranela R. Sodium selenite improves folliculogenesis in radiation-induced ovarian failure: a mechanistic approach. PloS One (2012) 7(12):e50928. doi: 10.1371/journal.pone.0050928 23236409 PMC3516513

[41] Borzoueisileh S Monfared AS Ghorbani H Mortazavi SMJ Zabihi E Pouramir M. Assessment of function, histopathological changes, and oxidative stress in liver tissue due to ionizing and non-ionizing radiations. Casp J Intern Med (2020) 11(3):315–23. doi: 10.22088/cjim.11.3.315 PMC7442457 32874440

[42] Abolfazl A Gholamali J Saeed N Tayyaba A Khadijeh N. Oxidative stress as the underlying biomechanism of detrimental outcomes of ionizing and non-ionizing radiation on human health: antioxidant protective strategies. Zahedan J Res Med Sci (2019) 21(4):1–9. doi: 10.5812/zjrms.85655

[43] Iuliis GND Newey RJ King BV Aitken RJ. Mobile phone radiation induces reactive oxygen species production and DNA damage in human spermatozoa *In Vitro* . PloS One (2009) 4(7):e6446. doi: 10.1371/journal.pone.0006446 19649291 PMC2714176

[44] Kesari KK Behari J. Evidence for mobile phone radiation exposure effects on reproductive pattern of male rats: role of ROS. Electromagn Biol Med (2012) 31(3):213–22. doi: 10.3109/15368378.2012.700292 22897402

[45] Pandey N Giri S. Melatonin attenuates radiofrequency radiation (900 MHz)-induced oxidative stress, DNA damage and cell cycle arrest in germ cells of male Swiss albino mice. Toxicol Ind Health (2018) 35(5):315–27. doi: 10.1177/0748233718758092 29562845

[46] Ji H Wang D Wu Y Niu Y Jia L Liu B. Wuzi Yanzong pill, a Chinese polyherbal formula, alleviates testicular damage in mice induced by ionizing radiation. BMC Complem Altern M (2016) 16(1):509. doi: 10.1186/s12906-016-1481-6 PMC5142375 27927244

[47] Houston BJ Nixon B King BV Aitken RJ Iuliis GND. Probing the origins of 1,800 MHz radio frequency electromagnetic radiation induced damage in mouse immortalized germ cells and spermatozoa *in vitro* . Front Public Health (2018) 6:270. doi: 10.3389/fpubh.2018.00270 30298125 PMC6160547

[48] Matsunuma R Niida H Ohhata T Kitagawa K Kitagawa M. UV Damage-induced phosphorylation of HBO1 triggers CRL4DDB2-mediated degradation to regulate cell proliferation. Mol Cell Biol (2016) 36(3):394–406. doi: 10.1128/MCB.00809-15 26572825 PMC4719422

[49] Aguilar-Mahecha A Hales BF Robaire B. Effects of acute and chronic cyclophosphamide treatment on meiotic progression and the induction of DNA double-strand breaks in rat spermatocytes. Biol Reprod (2005) 72(6):1297–304. doi: 10.1095/biolreprod.104.038620 15673603

[50] Jambor T Greifova H Kovacik A Kovacikova E Massanyi P Forgacs Z. Identification of *in vitro* effect of 4-octylphenol on the basal and human chorionic gonadotropin (hCG) stimulated secretion of androgens and superoxide radicals in mouse Leydig cells. J Environ Sci Heal (2019) 54(7-8):759–67. doi: 10.1080/10934529.2019.1592533 30925854

[51] Jambor T Greifova H Kovacik A Kovacikova E Tvrda E Forgacs Z. Parallel effect of 4-octylphenol and cyclic adenosine monophosphate (cAMP) alters steroidogenesis, cell viability and ROS production in mice leydig cells. Chemosphere Oxford (2018) 199:747–54. doi: 10.1016/j.chemosphere.2018.02.013 29478761

[52] Hana G Tomáš J Katarína T Ivana J Nikola K Norbert L. Resveratrol attenuates hydrogen peroxide-induced oxidative stress in TM3 Leydig cells *in vitro* . J Environ Sci Heal A (2020) 55(5):585–95. doi: 10.1080/10934529.2020.1717899 32178576

[53] Liu Y Yuan C Chen S Zheng Y Zhang Y Gao J. Global and cyp19a1a gene specific DNA methylation in gonads of adult rare minnow gobiocypris rarus under bisphenol a exposure. Aquat Toxicol (2014) 156:10–6. doi: 10.1016/j.aquatox.2014.07.017 25125231

[54] León-Martínez L López-Mendoza C Figueroa-Terán Y Flores-Ramírez R Alcantara L. Detection of aflatoxin B1 adducts in mexican women with cervical lesions. World Mycotoxin J (2021) 14(3):327–37. doi: 10.3920/WMJ2020.2602

[55] Hausen H. Human genital cancer: synergism between two virus infections or synergism between a virus infection and initiating events? Lancet (1982) 320(8312):1370–2. doi: 10.1016/S0140-6736(82)91273-9 6129466

[56] Saleh RA Agarwal A Kandirali E Sharma RK Thomas AJ Nada EA. Leukocytospermia is associated with increased reactive oxygen species production by human spermatozoa. Fertil Steril (2002) 78(6):1215–24. doi: 10.1016/S0015-0282(02)04237-1 12477515

[57] Zheng W Pan S Wang G Wang Y Liu Q Gu J. Zearalenone impairs the male reproductive system functions via inducing structural and functional alterations of sertoli cells. Environ Toxicol Phar (2016) 42:146–55. doi: 10.1016/j.etap.2016.01.013 26851377

[58] Liu W Han R Wu H Han D. Viral threat to male fertility. Andrologia (2018) 50(11):e13140. doi: 10.1111/and.13140 30569651

[59] Dong W Lv H Wang Y Li X Li C Wang L. The effect of classical swine fever virus NS5A and NS5A mutants on oxidative stress and inflammatory response in swine testicular cells. Res Vet Sci (2017) 112:89–96. doi: 10.1016/j.rvsc.2017.01.007 28142057

[60] Sun S Dosterman M Li M. Tissue specificity of DNA damage response and tumorigenesis. Cancer Biol Med (2019) 16(3):396–414. doi: 10.20892/j.issn.2095-3941.2019.0097 31565474 PMC6743622

[61] Kumari S Rastogi RP Singh KL Singh SP Sinha RP. DNA Damage: detection strategies. Excli J (2008) 7:44–62. doi: 10.17877/DE290R-8293

[62] Tünde SR Mária K Milán BK Miklós M Balázs K. Changes of DNA damage effect of T-2 or deoxynivalenol toxins during three weeks exposure in common carp (Cyprinus carpio L.) revealed by LORD-Q PCR. Toxins (2021) 13(8):576. doi: 10.3390/TOXINS13080576 34437447 PMC8402481

[63] Jones G Cooper C Petersson K. O105 - flash mechanisms track (oral presentations) comet assay measures indicate lower DNA damage levels in whole blood pbls following *ex vivo* electron flash exposures over 0.25–1% oxygen. Physica Med (2022) 94(S):S55. doi: 10.1016/S1120-1797(22)01556-3

[64] Zhang H Guo Y Ma W Xue J Wang W Yuan Z. MGMT is down-regulated independently of promoter DNA methylation in rats with all-trans retinoic acidinduced spina bifida aperta. Neural Regener Res (2019) 14(02):361–8. doi: 10.4103/1673-5374.244799 PMC6301176 30531021

[65] Gao H Shang Z Chan SY Dongli MA. Recent advances in the use of the CRISPR-Cas system for the detection of infectious pathogens. J Zhejiang Univ-Sc B (2022) 23(11):881–98. doi: 10.1631/JZUS.B2200068 PMC9676091 36379609

[66] Yu K Qiao Z Song W Bi S. Correction to: DNA nanotechnology for multimodal synergistic theranostics. J Anal Test (2021) 5(4):1–6. doi: 10.1007/S41664-021-00190-Z

[67] Wang G Hallberg LM Saphier E Englander EW. Short interspersed DNA element-mediated detection of UVB-induced DNA damage and repair in the mouse genome, *in vitro*, and *in vivo* in skin. Mutat Res (1999) 433(3):147–57. doi: 10.1016/S0921-8777(99)00007-5 10343648

[68] Zheng J Li X Shan D Zhang H Guan D. DNA Degradation within mouse brain and dental pulp cells 72 hours postmortem. Neural Regener Res (2012) 7(4):290–4. doi: 10.3969/j.issn.1673-5374.2012.04.009 PMC4353102 25806071

[69] Kumar K Lewis S Vinci S Riera-Escamilla A Fino MG Tamburrino L. Evaluation of sperm DNA quality in men presenting with testicular cancer and lymphoma using alkaline and neutral Comet assays. Andrology (2018) 6(1):230–5. doi: 10.1111/andr.12429 28950441

[70] Lee Y Wang Q Shuryak I Brenner DJ Turner HC. Development of a high-throughput γ-H2AX assay based on imaging flow cytometry. Radiat Oncol (2019) 14(1):150. doi: 10.1186/s13014-019-1344-7 31438980 PMC6704696

[71] Liu X Li X Su Z Zhang J Li H Xie J. Novel y-chromosomal microdeletions associated with non-obstructive azoospermia uncovered by high throughput sequencing of sequence-tagged sites (STSs). Sci Rep (2016) 6(1):21831. doi: 10.1038/srep21831 26907467 PMC4764820

[72] Jasmin F Jörg H Hans-Joachim R Christian M Susanne K Daniel H. Methylation of CpG 5962 in L1 of the human papillomavirus 16 genome as a potential predictive marker for viral persistence: a prospective large cohort study using cervical swab samples. Cancer Med (2020) 9(3):1058–68. doi: 10.1002/cam4.2771 PMC6997067 31856411

[73] Spivak G. Nucleotide excision repair in humans. DNA Repair (2015) 36:13–8. doi: 10.1016/j.dnarep.2015.09.003 PMC4688078 26388429

[74] Ding X Zhang A Li C Ma L Tang S Wang Q. The role of H3K9me2-regulated base excision repair genes in the repair of DNA damage induced by arsenic in HaCaT cells and the effects of Ginkgo biloba extract intervention. Environ Toxicol (2020) 36(5):850–60. doi: 10.1002/TOX.23088 33378118

[75] Chandrima C Arup D Chandra B Shuddhanjali R Tanushree A Sudipta R. Chloroplastic RecA protein from physcomitrium patens is able to repair chloroplastic DNA damage by homologous recombination but unable to repair nuclear DNA damage. Physiol Mol Biol Pla (2022) 28(11-12):2057–67. doi: 10.1007/S12298-022-01264-7 PMC9789214 36573145

[76] Suwaki N Klare K Tarsounas M. RAD51 paralogs: roles in DNA damage signalling, recombinational repair and tumorigenesis. Semin Cell Dev Biol (2011) 22(8):898–905. doi: 10.1016/j.semcdb.2011.07.019 21821141

[77] Roger WLG Nicole V Marcha V Carole LY Joost OL Harry VS. Effects of benzo[a]pyrene on mouse germ cells: heritable DNA mutation, testicular cell hypomethylation and their interaction with nucleotide excision repair. Toxicol Res (2015) 4(3):718–24. doi: 10.1039/c4tx00114a

[78] Izumi T Wiederhold LR Roy G Roy R Jaiswal A Bhakat KK. Mammalian DNA base excision repair proteins: their interactions and role in repair of oxidative DNA damage. Toxicol (2003) 193(1):43–65. doi: 10.1016/S0300-483X(03)00289-0 14599767

[79] Yun-Jeong K Wilson DM. Overview of base excision repair biochemistry. Curr Mol Pharmacol (2012) 5(1):3–13. doi: 10.2174/1874467211205010003 22122461 PMC3459583

[80] Wallace SS Murphy DL Sweasy JB. Base excision repair and cancer. Cancer Lett (2012) 327(1-2):73–89. doi: 10.1016/j.canlet.2011.12.038 22252118 PMC3361536

[81] Gunes S Al-Sadaan M Agarwal A. Spermatogenesis, DNA damage and DNA repair mechanisms in male infertility. Reprod BioMed Online (2015) 31(3):309–19. doi: 10.1016/j.rbmo.2015.06.010 26206278

[82] Bee L Fabris S Cherubini R Mognato M Celotti L. The efficiency of homologous recombination and non-homologous end joining systems in repairing double-strand breaks during cell cycle progression. PloS One (2013) 8(7):e69061. doi: 10.1371/journal.pone.0069061 23874869 PMC3708908

[83] Orhan E Velazquez C Tabet I Sardet C Theillet C. Regulation of RAD51 at the transcriptional and functional levels: what prospects for cancer therapy? Cancers (2021) 13(12):2930. doi: 10.3390/CANCERS13122930 34208195 PMC8230762

[84] Bertelsen B Tuxen IV Yde CW Gabrielaite M Nielsen FC. High frequency of pathogenic germline variants within homologous recombination repair in patients with advanced cancer. NPJ Genom Med (2019) 4(1):1–11. doi: 10.1038/s41525-019-0087-6 31263571 PMC6588611

[85] Caggiano C Cavallo F Giannattasio T Cappelletti G Rossi P Grimaldi P. Testicular germ cell tumors acquire cisplatin resistance by rebalancing the usage of DNA repair pathways. Cancers (2021) 13(4):787. doi: 10.3390/CANCERS13040787 33668653 PMC7917736

[86] Liu K Mao X Pan F An RF. Effect and mechanisms of reproductive tract infection on oxidative stress parameters, sperm DNA fragmentation, and semen quality in infertile males. Reprod Biol Endocrin (2021) 19(1):97. doi: 10.1186/S12958-021-00781-6 PMC8237428 34183027

[87] Mauri AL Vagnini LD Petersen CG Oliveira J Baruffi R Franco JG. An evaluation of the effect of herpesvirus-associated ubiquitin-specific protease (HAUSP) gene polymorphism on etiopathogenic factors of DNA damage in sperm. Fertility Sterility (2013) 100(3S):S441. doi: 10.1016/j.fertnstert.2013.07.563

[88] Aboulmaouahib S Madkour A Kaarouch I Sefrioui O Saadani B Copin H. Impact of alcohol and cigarette smoking consumption in male fertility potential: looks at lipid peroxidation, enzymatic antioxidant activities and sperm DNA DNA damage. Andrologia (2018) 50(3):1. doi: 10.1111/and.12926 29164649

[89] Hewedy S Hassan G Enein R Saad M. Sperm DNA damage in smokers. Hum Androl (2017) 7(2):65–72. doi: 10.21608/ha.2017.1172.1005

[90] Herati AS Zhelyazkova BH Butler PR Lamb DJ. Age-related alterations in the genetics and genomics of the male germ line. Fertil Steril (2017) 107(2):319–23. doi: 10.1016/j.fertnstert.2016.12.021 28160920

[91] Das M Al-Hathal N San-Gabriel M Phillips S Zini A. High prevalence of isolated sperm DNA damage in infertile men with advanced paternal age. J Assist Reprod Gen (2013) 30(6):843–48. doi: 10.1007/s10815-013-0015-0 PMC3696445 23722935

[92] Wyrobek AJ Eskenazi B Young S Arnheim N Tiemann-Boege I Jabs E. Advancing age has differential effects on DNA damage, chromatin integrity, gene mutations, and aneuploidies in sperm. P Natl Acad Sci USA (2006) 103(25):9601–06. doi: 10.1073/pnas.0506468103 PMC1480453 16766665

[93] Templado C Donate A Giraldo J Bosch M Estop A. Advanced age increases chromosome structural abnormalities in human spermatozoa. Eur J Hum Genet (2011) 19(2):145–51. doi: 10.1038/ejhg.2010.166 PMC3025794 21045871

[94] Gao Z Wyman MJ Sella G Przeworski M. Interpreting the dependence of mutation rates on age and time. PloS Biol (2016) 14(1):e1002355. doi: 10.1371/journal.pbio.1002355 26761240 PMC4711947

[95] Ségurel L Wyman MJ Przeworski M. Determinants of mutation rate variation in the human germline. Annu Rev Genom Hum G (2014) 15(1):47–70. doi: 10.1146/annurev-genom-031714-125740 25000986

[96] Aylwyn S. Mutation rates and the evolution of germline structure. Philos T R Soc B (2016) 37(1699):1–10. doi: 10.1098/rstb.2015.0137 PMC4920338 27325834

[97] Bommarito PA Fry RC. The role of DNA methylation in gene regulation. Toxicoepigenetics (2019), 127–51. doi: 10.1016/B978-0-12-812433-8.00005-8

[98] Saleh RA Agarwal A Nada EA El-Tonsy MH Sharma RK Meyer A. Negative effects of increased sperm DNA damage in relation to seminal oxidative stress in men with idiopathic and male factor infertility. Fertil Steril (2003) 79(supp-S3):1597–605. doi: 10.1016/S0015-0282(03)00337-6 12801566

[99] Abd-Elmoaty MA Saleh R Sharma R Agarwal A. Increased levels of oxidants and reduced antioxidants in semen of infertile men with varicocele. Fertil Steril (2010) 94(4):1531–34. doi: 10.1016/j.fertnstert.2009.12.039 20117772

[100] Agarwal A Said TM. Role of sperm chromatin abnormalities and DNA damage in male infertility. Hum Reprod Update (2003) 9(4):331–45. doi: 10.1093/humupd/dmg027 12926527

[101] Aitken RJ Iuliis G. Origins and consequences of DNA damage in male germ cells. Reprod BioMed Online (2007) 14(6):727–33. doi: 10.1016/S1472-6483(10)60676-1 17579989

[102] Aitken RJ De Iuliis GN McLachlan RI. Biological and clinical significance of DNA damage in the male germ line. Int J Androl (2009) 32(1):46–56. doi: 10.1111/j.1365-2605.2008.00943.x 19076252

[103] van Overveld FWPC Haenen GRMM Rhemrev J Vermeiden JPW Bast A. Tyrosine as important contributor to the antioxidant capacity of seminal plasma. Chem-Biol Interact (2000) 127(2):151–61. doi: 10.1016/S0009-2797(00)00179-4 10936230

[104] Simon L Emery BR Carrell DT. Review: impact of sperm DNA damage in assisted reproduction. Best Pract Res Cl Ob (2017) 44:38–56. doi: 10.1016/j.bpobgyn.2017.07.003 28935366

[105] Collodel G Moretti E Micheli L Menchiari A Moltoni L Cerretani D. Semen characteristics and malondialdehyde levels in men with different reproductive problems. Andrology-Us (2015) 3(2):280–6. doi: 10.1111/andr.297 25331426

[106] El-Mohamady RS Behour TS Rawash ZM. Concurrent detection of bovine viral diarrhoea virus and bovine herpesvirus-1 in bulls’ semen and their effect on semen quality. Int J Vet Sci Med (2020) 8(1):106–14. doi: 10.1080/23144599.2020.1850197 PMC7751410 33426047

[107] Tremellen K Tunc O. Macrophage activity in semen is significantly correlated with sperm quality in infertile men. Int J Androl (2010) 33(6):823–31. doi: 10.1111/j.1365-2605.2009.01037.x 20132344

[108] Plessis S Agarwal A Halabi J Tvrda E. Contemporary evidence on the physiological role of reactive oxygen species in human sperm function. J Assist Reprod Gen (2015) 32(4):509–20. doi: 10.1007/s10815-014-0425-7 PMC4380893 25646893

[109] Pasqualotto FF Sharma RK Potts JM Nelson DR Thomas AJ Agarwal A. Seminal oxidative stress in patients with chronic prostatitis. Urology (2000) 35(5):304–08. doi: 10.1111/j.1439-0272.2003.tb00862.x 10840100

[110] Agarwal A Mulgund A Alshahrani S Assidi M Sabanegh E. Reactive oxygen species and sperm DNA damage in infertile men presenting with low level leukocytospermia. Reprod Biol Endocrin (2014) 12(1):126. doi: 10.1186/1477-7827-12-126 PMC4292986 25527074

[111] Vicari E. Seminal leukocyte concentration and related specific reactive oxygen species production in patients with male accessory gland infections. Hum Reprod (1999) 14(8):2025–30. doi: 10.1093/humrep/15.12.2536 10438421

[112] Gallegos G Ramos B Santiso R Goyanes V Gosálvez J Luis JF. Sperm DNA fragmentation in infertile men with genitourinary infection by Chlamydia trachomatis and Mycoplasma. Fertil Steril (2008) 90(2):328–34. doi: 10.1016/j.fertnstert.2007.06.035 17953955

[113] Yadav A Rene ER Mandal MK Dubey KK. Threat and sustainable technological solution for antineoplastic drugs pollution: review on a persisting global issue. Chemosphere (2020) 263(2):128285. doi: 10.1016/j.chemosphere.2020.128285 33297229

[114] Kovacik A Arvay J Tusimova E Harangozo L Tvrda E Zbynovska K. Seasonal variations in the blood concentration of selected heavy metals in sheep and their effects on the biochemical and hematological parameters. Chemosphere (2017) 168:365–71. doi: 10.1016/j.chemosphere.2016.10.0901 27810536

[115] Tung CK Lin C Harvey B Fiore AG Ardon F Wu M. Fluid viscoelasticity promotes collective swimming of sperm. Sci Rep-Uk (2017) 7(1):3152. doi: 10.1038/s41598-017-03341-4 PMC5466690 28600487

[116] Atig F Raffa M Ali HB Abdelhamid K Saad A Ajina M. Altered antioxidant status and increased lipid per-oxidation in seminal plasma of tunisian infertile men. Int J Biol Sci (2012) 8(1):139–49. doi: 10.7150/IJBS.8.139 PMC3248656 22211112

[117] Juyena NS Stelletta C. Seminal plasma: an essential attribute to spermatozoa review. J Androl (2012) 33(4):536–51. doi: 10.2164/jandrol.110.012583 22016346

[118] Zhou N Jiang C Chen Q Yang H Wang X Zou P. Exposures to atmospheric PM_10_ and PM_10-2.5_ affect male semen quality: results of MARHCS study. Environ Sci Technol (2018) 52(3):1571–81. doi: 10.1021/acs.est.7b05206 29320852

[119] Anderson D Schmid TE Baumgartner A. Paternal smoking as a cause for transgenerational damage in the offspring. Handb Fertil (2015), 19–26. doi: 10.1016/B978-0-12-800872-0.00002-0

[120] Malek MA Dasiman R Khan N Mohamed-Akhlak S Mahmud M. The protective effects of procyanidin C-1 on bisphenol A-induced testicular dysfunction in aged mice. Food Sci Hum Well (2022) 11(4):965–74. doi: 10.1016/j.fshw.2022.03.020

[121] Plaseski T Noveski P Popeska Z Efremov GD Plaseska-Karanfilska D. Association study of single-nucleotide polymorphisms in FASLG, JMJDIA, LOC203413, TEX15, BRDT, OR2W3, INSR, and TAS2R38 genes with male infertility. J Androl (2012) 33(4):675–83. doi: 10.2164/jandrol.111.013995 22016351

[122] Chen K Mai Z Zhou Y Gao X Yu B. Low NRF2 mRNA expression in spermatozoa from men with low sperm motility. Tohoku J Exp Med (2012) 228(3):259–66. doi: 10.1620/tjem.228.259 23089668

[123] Siddique RA Jagan MG Raj K Anand K Malik PK Chandan K. Sperm abnormalities and DNA fragmentation vis-à-vis mammalian male infretility – a review. Wayamba J Anim Sci (2011), 174–89.

[124] Varshini J Srinag BS Kalthur G Krishnamurthy H Kumar P Rao BS. Poor sperm quality and advancing age are associated with increased sperm DNA damage in infertile men. Andrologia (2012) 44(s1):642–49. doi: 10.1111/j.1439-0272.2011.01243.x 22040161

[125] Simon L Emery B Carrell DT. Sperm DNA fragmentation: consequences for reproduction. Adv Exp Med Biol (2019) 1166:87–105. doi: 10.1007/978-3-030-21664-1_6 31301048

[126] Mayer A Baran V Sakakibara Y Brzakova A Ferencova I Motlik J. DNA Damage response during mouse oocyte maturation. Cell Cycle (2016) 15(4):546–58. doi: 10.1080/15384101.2015.1128592 PMC5056612 26745237

[127] Zhang D Zhang X Zeng M Yuan J Liu M Yin Y. Increased DNA damage and repair deficiency in granulosa cells are associated with ovarian aging in rhesus monkey. J Assist Reprod Genet (2015) 32(7):1069–78. doi: 10.1007/s10815-015-0483-5 PMC4531862 25957622

[128] Oktay K Turan V Titus S Stobezki R Liu L. *Biol R* BRCA mutations, DNA repair deficiency, and ovarian aging. Biol Reprod (2015) 93(3):67. doi: 10.1095/biolreprod.115.132290 26224004 PMC4710189

[129] Wayne L Shiny T Fred M GE S Kutluk O. Ovarian aging in women with BRCA germline mutations. J Clin Endocrinol Metab (2017) 102(10):3839–47. doi: 10.1210/jc.2017-00765 PMC5630253 28938488

[130] Wesevich V Kellen AN Pal L. Recent advances in understanding primary ovarian insufficiency. F1000Res (2020) 9:F1000 Faculty Rev–1101. doi: 10.12688/f1000research.26423.1 PMC7477642 32934798

[131] Han L Ren C Li L Li X Ge J Wang H. Embryonic defects induced by maternal obesity in mice derive from Stella insufficiency in oocytes. Nat Genet (2018) 50(3):432–42. doi: 10.1038/s41588-018-0055-6 29459681

[132] Wang L Chen Y Wei J Guo F Li L Han Z. Administration of nicotinamide mononucleotide improves oocyte quality of obese mice. Cell Proliferat (2022) 55(11):e13303. doi: 10.1111/CPR.13303 PMC9628229 35811338

[133] Avdatek F Birdane YO Türkmen R Demirel HH. Ameliorative effect of resveratrol on testicular oxidative stress, spermatological parameters and DNA damage in glyphosate-based herbicide-exposed rats. Andrologia (2018):e13036. doi: 10.1111/and.13036 29761542

[134] Jager CD Farias P Barraza-Villarreal A Avila MH Bailey JL. Reduced seminal parameters associated with environmental DDT exposure and p,p '-DDE concentrations in men in Chiapas, Mexico: a cross-sectional study. J Androl (2006) 27(1):16–27. doi: 10.2164/jandrol.05121 16400073

[135] Xia D Parvizi N Zhou Y Xu K Jiang H Li R. Paternal fenvalerate exposure influences reproductive functions in the offspring. Reprod Sci (2013) 20(11):1308–15. doi: 10.1177/1933719113483015 PMC3795420 23548413

[136] Gadah A Tarfa A Nouf A Saleh A AM S KR B. Red beetroot extract mitigates chlorpyrifos-induced reprotoxicity associated with oxidative stress, inflammation, and apoptosis in rats. Environ Sci pollut R (2020) 27(4):3979–91. doi: 10.1007/s11356-019-07009-6 31823260

[137] Sadakierska-Chudy A Patrylak J Janeczko J Chudy J. Downregulation of gene expression and the outcome of ICSI in severe oligozoospermic patients: a preliminary study. Mol Reprod Dev (2020) 87(12):1219–30. doi: 10.1002/mrd.23442 33241638

[138] Abd-Elrazek AM El-Dash HA Said NI. The role of propolis against paclitaxel-induced oligospermia, sperm abnormality, oxidative stress and DNA damage in testes of male rats. Andrologia (2020) 52(1):e13394. doi: 10.1111/and.13394 31762066

[139] Ahmed TA Pallav S Sulagna D Aldo EC. Coenzyme Q10, oxidative stress markers, and sperm DNA damage in men with idiopathic oligoasthenoteratospermia. Clin Exp Reprod Med (2021) 48(2):150–5. doi: 10.5653/cerm.2020.04084 PMC8176152 34078008

[140] Zaki M Basha W El-Bassyouni HT El-Toukhy S Hussein T. Evaluation of DNA damage profile in obese women and its association to risk of metabolic syndrome, polycystic ovary syndrome and recurrent preeclampsia. Genes Dis (2018) 5(4):367–73. doi: 10.1016/j.gendis.2018.03.001 PMC6303482 30591939

[141] Heddar A Guichoux N Auger N Misrahi M. P–577 impaired double strand DNA repair in isolated primary ovarian insufficiency with homozygous nonsense mutation of SPIDR. Hum Reprod (2021) 36(Supplement_1):deab130.576. doi: 10.1093/humrep/deab130.576

[142] Marc DB Huong NM Frédéric M Aurore P. Genetic aspects of monomorphic teratozoospermia: a review. J Assist Reprod Gen (2015) 32(4):615–23. doi: 10.1007/s10815-015-0433-2 PMC4380889 25711835

[143] Zhou Y Chen Y Hu X Guo J Shi H Yu G. Icariin attenuate microcystin-LR-induced gap junction injury in sertoli cells through suppression of akt pathways. Environ pollut (2019) 251:328–37. doi: 10.1016/j.envpol.2019.04.114 31091496

[144] Rao MV Shah NP Raval RJ Solanki PP Shah SC. Current status of y chromosome microdeletions: prevalence, distribution, implication and association with Male infertility in Indian men- a review. J Clin Diagn Res (2021) 15(3):GE01–9. doi: 10.7860/JCDR/2021/46154.14604

[145] Carto C Gandhi DA Nackeeran S Madhusoodanan V Ramasamy R. Varicocele is underdiagnosed in men evaluated for infertility: examination of multi-center large-scale electronic health record data. Andrologia (2022) 54(10):e14539. doi: 10.1111/and.14539 35914741

[146] Zhang QF Wang S Zhang H Liu QL Wei Y Deng W. Effects of alpha-lipoic acid on sperm quality in patients with varicocele-related male infertility: study protocol for a randomized controlled clinical trial. Trials (2022) 23(1):1002. doi: 10.1186/s13063-022-06951-0 36510262 PMC9746131

[147] Park YJ Pang MG. Mitochondrial functionality in Male fertility: from spermatogenesis to fertilization. Antioxidants (Basel) (2021) 10(1):98. doi: 10.3390/antiox10010098 33445610 PMC7826524

[148] Zhang Z Yang Q. The testosterone mimetic properties of icariin. Asian J andrology (2006) 8(5):601–5. doi: 10.1111/j.1745-7262.2006.00197.x 16751992

[149] Verit FF Erel O. Oxidative stress in nonobese women with polycystic ovary syndrome: correlations with endocrine and screening parameters. Gynecol Obstet Invest (2008) 65(4):233–9. doi: 10.1159/000113046 18196905

[150] Showell MG Brown J Yazdani A Stankiewicz MT Hart RJ. Antioxidants for male subfertility. Cochrane Db Syst Rev (2019) 3(1):CD007411. doi: 10.1002/14651858.CD007411.pub2 PMC6416049 30866036

[151] Caburet S Arboleda VA Llano E Overbeek PA Barbero JL Oka K. Mutant cohesin in premature ovarian failure. N Engl J Med (2014) 370(10):943–9. doi: 10.1056/NEJMoa1309635 PMC4068824 24597867

[152] Winters T Mcnicoll F Jessberger R. Meiotic cohesin STAG3 is required for chromosome axis formation and sister chromatid cohesion. EMBO J (2014) 33(11):1256–70. doi: 10.1002/embj.201387330 PMC4198028 24797474

[153] Phil VN Peter NS. Sperm DNA damage and its role in IVF and ICSI. Basic Clin Androl (2016) 26:15. doi: 10.1186/s12610-016-0043-6 27980786 PMC5137216

[154] Olsen AK Lindeman B Wiger R Duale N Brunborg G. How do male germ cells handle DNA damage? Toxicol Appl Pharmacol (2005) 207(2-supp-S):521–31. doi: 10.1016/j.taap.2005.01.060 16051290

[155] Houda G Asma B Stéphane V Ali S Samira IR. Comparison of sperm morphology and nuclear sperm quality in SPATA16- and DPY19L2-mutated globozoospermic patients. Andrologia (2019) 51(6):e13277. doi: 10.1111/and.13277 30912172

[156] Koscinski I Elinati E Fossard C Redin C Viville S Calle JV. DPY19L2 deletion as a major cause of globozoospermia. Am J Hum Genet (2011) 88(3):344–50. doi: 10.1016/j.ajhg.2011.03.014 PMC3059416 21397063

[157] Hargreave TB. Genetic basis of Male infertility. Brit Med Bull (2000) 56(3):650–71. doi: 10.1258/0007142001903454 11255552

[158] Tiepolo L Zuffardi O. Localization of factors controlling spermatogenesis in the nonfluorescent portion of the human y chromosome long arm. Hum Genet (1976) 34(2):119–24. doi: 10.1007/BF00278879 1002136

[159] Feugang JM Youngblood RC Greene JM Fahad AS Monroe WA Willard ST. Application of quantum dot nanoparticles for potential non-invasive bio-imaging of mammalian spermatozoa. J Nanobiotechnology (2012) 10:45. doi: 10.1186/1477-3155-10-45 23241497 PMC3553073

[160] He W Liu H Hu L Wang Y Huang L Liang A. Icariin improves testicular dysfunction via enhancing proliferation and inhibiting mitochondria-dependent apoptosis pathway in high-fat diet and streptozotocin-induced diabetic rats. Reprod Biol Endocrin (2021) 19(1):168. doi: 10.1186/s12958-021-00851-9 PMC8576896 34753504

[161] Kao SH Chao HT Wei YH. Multiple deletions of mitochondrial DNA are associated with the decline of motility and fertility of human spermatozoa. Mol Hum Reprod (1998) 4(7):657–66. doi: 10.1093/molehr/4.7.657 9701788

[162] Héctor F-EM. Polycystic ovary syndrome: definition, aetiology, diagnosis and treatment. Nat Rev Endocrinol (2018) 14(5):270–84. doi: 10.1038/nrendo.2018.24 29569621

[163] Randeva HS Tan BK Weickert MO Lois K Nestler JE Sattar N. Cardiometabolic aspects of the polycystic ovary syndrome. Endocr Rev (2012) 33(5):812–41. doi: 10.1210/er.2012-1003 PMC3461136 22829562

[164] Liu Y Yu Z Zhao S Cheng L Man Y Gao X. Oxidative stress markers in the follicular fluid of patients with polycystic ovary syndrome correlate with a decrease in embryo quality. J Assist Reprod Genet (2021) 38(2):471–7. doi: 10.1007/s10815-020-02014-y PMC7884504 33216309

[165] Chen L Xu WM Zhang D. Association of abdominal obesity, insulin resistance, and oxidative stress in adipose tissue in women with polycystic ovary syndrome. Fertil Steril (2014) 102(4):1167–74.e4. doi: 10.1016/j.fertnstert.2014.06.027 25064406

[166] Shan HY Luo RX Guo XY Li R Ye ZH Peng TL. Abnormal endometrial receptivity and oxidative stress in polycystic ovary syndrome. Front Pharmaco (2022) 13:904942. doi: 10.3389/fphar.2022.904942 PMC9357999 35959444

[167] Dumesic DA Meldrum DR Katz-Jaffe MG Krisher RL Schoolcraft WB. Oocyte environment: follicular fluid and cumulus cells are critical for oocyte health. Fertil Steril (2015) 103(2):303–16. doi: 10.1016/j.fertnstert.2014.11.015 25497448

[168] Özer A Bakacak M Kıran H Ercan Ö Köstü B Kanat-Pektaş M. Increased oxidative stress is associated with insulin resistance and infertility in polycystic ovary syndrome. Ginekol Pol (2016) 87(11):733–8. doi: 10.5603/gp.2016.0079 27958630

[169] Huhtaniemi I Hovatta O Marca AL Livera G Monniaux D Persani L. Advances in the molecular pathophysiology, genetics, and treatment of primary ovarian insufficiency. Trends Endocrinol Metab (2018) 29(6):400–19. doi: 10.1016/j.tem.2018.03.010 29706485

[170] Yang X Zhang XJ Jiao J Zhang F Pan YC Wang QQ. Rare variants in FANCA induce premature ovarian insufficiency. Hum Genet (2019) 138(11-12):1227–36. doi: 10.1007/s00439-019-02059-9 PMC6874525 31535215

[171] Vabre P Gatimel N Moreau J Gayrard V Picard-Hagen N Parinaud J. Environmental pollutants, a possible etiology for premature ovarian insufficiency: a narrative review of animal and human data. Environ Health (2017) 16(1):37. doi: 10.1186/s12940-017-0242-4 28388912 PMC5384040

[172] Pu D Wang C Cao J Shen Y Jiang H Liu J. Association analysis between HFM1 variation and primary ovarian insufficiency in Chinese women. Clin Genet (2016) 89(5):597–602. doi: 10.1111/cge.12718 26679638

[173] Li N Wang J Wang X Sun J Li Z. Icariin exerts a protective effect against d-galactose induced premature ovarian failure via promoting DNA damage repair. BioMed Pharmacother (2019) 118:109218. doi: 10.1016/j.biopha.2019.109218 31330441

[174] Pini T Makloski R Maruniak K Schoolcraft WB Katz-Jaffe MG. Mitigating the effects of oxidative sperm DNA damage. Antioxidants (Basel) (2020) 9(7):589. doi: 10.3390/antiox9070589 32640607 PMC7402125

[175] Yin L Liu Y Peng Y Peng Y Yu X Gao Y. Correction to: PARP inhibitor veliparib and HDAC inhibitor SAHA synergistically co-target the UHRF1/ BRCA1 DNA damage repair complex in prostate cancer cells. J Exp Clin Cancer Res (2018) 37(1):153. doi: 10.1186/s13046-018-0810-7 30012171 PMC6048811

[176] Asgari F Gavahi A Karimi M Vatannejad A Amjadi F Aflatoonian R. Risk of embryo aneuploidy is affected by the increase in sperm DNA damage in recurrent implantation failure patients under ICSI-CGH array cycles. Hum Fertil (Camb) (2021) 25(5):872–80. doi: 10.1080/14647273.2021.1920054 33938375

[177] Rychtarova J Langerova A Fulka H Loi P Benc M Fulka JJ. Interspecific ICSI for the assessment of sperm DNA damage: technology report. Animals-Basel (2021) 11(5):1250. doi: 10.3390/ani11051250 33926086 PMC8145464

[178] Zhai Y Guo X Pan Y Niu Y Li C Wu X. A systematic review of the efficacy and pharmacological profile of Herba Epimedii in osteoporosis therapy. Pharmazie (2013) 68(9):713–22. doi: 10.1691/ph.2013.2900 24147339

[179] Chen M Hao J Yang Q Li G. Effects of icariin on reproductive functions in Male rats. Molecules (2014) 19(7):9502–14. doi: 10.3390/molecules19079502 PMC6271987 24995929

[180] Nan Y Zhang X Yang G Xie J Lu Z Wang W. Icariin stimulates the proliferation of rat Sertoli cells in an ERK1/2-dependent manner *in vitro* . Andrologia (2014) 46(1):9–16. doi: 10.1111/and.12035 23134192

[181] Scaruffi P Licata E Maccarini E Massarotti C Bovis F Sozzi F. Oral antioxidant treatment of men significantly improves the reproductive outcome of IVF cycles. J Clin Med (2021) 10(15):3254. doi: 10.3390/jcm10153254 34362038 PMC8347466

[182] Pothuri B O'Cearbhaill R Eskander R Armstrong D. Frontline PARP inhibitor maintenance therapy in ovarian cancer: a society of gynecologic oncology practice statement. Gynecol Oncol (2020) 159(1):8–12. doi: 10.1016/j.ygyno.2020.07.097 32778410

[183] Cheng H Yang J Liu H Xiang Y. Poly (adenosine diphosphate [ADP]–ribose) polymerase (PARP) inhibitors as maintenance therapy in women with newly diagnosed ovarian cancer: a systematic review and meta-analysis. Arch Gynecol Obstet (2021) 304(2):285–96. doi: 10.1007/s00404-021-06070-2 PMC8277645 34021367

[184] Barkalina N Charalambous C Jones C Coward K. Nanotechnology in reproductive medicine: emerging applications of nanomaterials. Nanomedicine (2014) 10(5):921–38. doi: 10.1016/j.nano.2014.01.001 24444494

[185] Rath D Barcikowski S de Graaf S Garrels W Grossfeld R Klein S. Sex selection of sperm in farm animals: status report and developmental prospects. Reproduction (2015) 145(1):R15–30. doi: 10.1530/REP-12-0151 23148085

[186] Fynewever TL Agcaoili ES Jacobson JD Patton WC Chan PJ. *In vitro* tagging of embryos with nanoparticles. J Assist Reprod Genet (2007) 24(2-3):61–5. doi: 10.1007/s10815-006-9084-7 PMC3454985 17195099

[187] Makhluf SB Abu-Mukh R Rubinstein S Breitbart H Gedanken A. Modified PVA–Fe3O4 nanoparticles as protein carriers into sperm cells. Small (2008) 4(9):1453–8. doi: 10.1002/smll.200701308 18680094

[188] Kim TS Lee SH Gang GT Lee YS Kim SU Koo DB. Exogenous DNA uptake of boar spermatozoa by a magnetic nanoparticle vector system. Reprod Domest Anim (2010) 45(5):e201–6. doi: 10.1111/j.1439-0531.2009.01516.x 19788517

[189] Dai P Liu C Xie C Ke J He Y Wei L. TiO2 nanotubes loaded with CdS nanocrystals as enhanced emitters of electrochemiluminescence: application to an assay for prostate-specific antigen. Anal Bioanal Chem (2020) 412(6):1375–84. doi: 10.1007/s00216-019-02365-1 31919610

[190] Hee-Man Y Woo PC Min-Ah W Il KM Min JY Gyu PH. HER2/neu antibody conjugated poly(amino acid)-coated iron oxide nanoparticles for breast cancer MR imaging. Biomacromolecules (2010) 11(11):2866–72. doi: 10.1021/bm100560m 20932000

[191] Zillhardt M Christensen JG Lengyel E. An orally available small-molecule inhibitor of c-Met, PF-2341066, reduces tumor burden and metastasis in a preclinical model of ovarian cancer aetastasis. Neoplasia (2010) 12(1):1–10. doi: 10.1593/neo.09948 20072648 PMC2805878

[192] Jin X Gossett DR Wang S Yang D Cao Y Chen J. Inhibition of AKT survival pathway by a small molecule inhibitor in human endometrial cancer cells. Brit J Cancer (2004) 91(10):1808–12. doi: 10.1038/SJ.BJC.6602214 PMC2410058 15505622

[193] Wang S Tan W Huang Y Mao X Xue L. Sperm DNA fragmentation measured by sperm chromatin dispersion impacts morphokinetic parameters, fertilization rate and blastocyst quality in ICSI treatments. Zygote (2021) 30(1):72–9. doi: 10.1017/S0967199421000332 34034847

[194] Lara-Cerrillo S Ribas-Maynou J Rosado-Iglesias C Lacruz-Ruiz T Benet J García-Peiró A. Sperm selection during ICSI treatments reduces single- but not double-strand DNA break values compared to the semen sample. J Assist Reprod Gen (2021) 38(5):1187–96. doi: 10.1007/s10815-021-02129-w PMC8190426 33660206

[195] Pourmand G Movahedin M Noori M Dehghani S Hosseini M Ziloochi M. Does antioxidant therapy add any benefit in improvement of DNA damage to standard inguinal varicocelectomy? a randomized case-control study. Eur Urol Suppl (2014) 13(1):e251. doi: 10.1016/S1569-9056(14)60247-5

[196] Nagano M Shinohara T Avarbock MR Brinster RL. Retrovirus-mediated gene delivery into male germ line stem cells. FEBS Lett (2000) 475(1):7–10. doi: 10.1016/s0014-5793(00)01606-9 10854847

[197] Rahbar M Asadpour R Azami M Mazaheri Z Hamali H. Improving the process of spermatogenesis in azoospermic mice using spermatogonial stem cells co-cultured with epididymosomes in three-dimensional culture system. Life Sci (2022) 310:121057. doi: 10.1016/j.lfs.2022.121057 36220369

[198] Huang B Ding C Zou Q Lu J Wang W Li H. Human amniotic fluid mesenchymal stem cells improve ovarian function during physiological aging by resisting DNA damage. Front Pharmacol (2020) 11:272. doi: 10.3389/fphar.2020.00272 32273842 PMC7113373

